# Genetic Loci Governing Grain Yield and Root Development under Variable Rice Cultivation Conditions

**DOI:** 10.3389/fpls.2017.01763

**Published:** 2017-10-16

**Authors:** Margaret Catolos, Nitika Sandhu, Shalabh Dixit, Noraziya A. A. Shamsudin, Ma E. B. Naredo, Kenneth L. McNally, Amelia Henry, Ma G. Diaz, Arvind Kumar

**Affiliations:** ^1^Rice Breeding Platform, International Rice Research Institute, Manila, Philippines; ^2^Genetics and Molecular Biology Division, Institute of Biological Sciences, University of the Philippines Los Baños, Los Baños, Philippines; ^3^School of Environmental and Natural Resource Sciences, Universiti Kebangsaan Malaysia, Bangi, Malaysia

**Keywords:** drought, food security, grain yield, QTL, rice, root traits

## Abstract

Drought is the major abiotic stress to rice grain yield under unpredictable changing climatic scenarios. The widely grown, high yielding but drought susceptible rice varieties need to be improved by unraveling the genomic regions controlling traits enhancing drought tolerance. The present study was conducted with the aim to identify quantitative trait loci (QTLs) for grain yield and root development traits under irrigated non-stress and reproductive-stage drought stress in both lowland and upland situations. A mapping population consisting of 480 lines derived from a cross between Dular (drought-tolerant) and IR64-21 (drought susceptible) was used. QTL analysis revealed three major consistent-effect QTLs for grain yield (*qDTY*_*1.1*_*, qDTY*_*1.3*_, and *qDTY*_*8.1*_) under non-stress and reproductive-stage drought stress conditions, and 2 QTLs for root traits (*qRT*_*9.1*_ for root-growth angle and *qRT*_*5.1*_ for multiple root traits, i.e., seedling-stage root length, root dry weight and crown root number). The genetic locus *qDTY*_*1.1*_ was identified as hotspot for grain yield and yield-related agronomic and root traits. The study identified significant positive correlations among numbers of crown roots and mesocotyl length at the seedling stage and root length and root dry weight at depth at later stages with grain yield and yield-related traits. Under reproductive stage drought stress, the grain yield advantage of the lines with QTLs ranged from 24.1 to 108.9% under upland and 3.0–22.7% under lowland conditions over the lines without QTLs. The lines with QTL combinations *qDTY*_*1.3*_+*qDTY*_*8.1*_ showed the highest mean grain yield advantage followed by lines having *qDTY*_*1.1*_+*qDTY*_*8.1*_ and *qDTY*_*1.1*_+*qDTY*_*8.1*_+*qDTY*_*1.3*_, across upland/lowland reproductive-stage drought stress. The identified QTLs for root traits, mesocotyl length, grain yield and yield-related traits can be immediately deployed in marker-assisted breeding to develop drought tolerant high yielding rice varieties.

## Introduction

The development of rice cultivars with improved tolerance for drought stress is important to increase the production of rainfed rice ecosystems. Efficient use of existing genetic variability in traditional cultivars by employing advanced tools and techniques is needed to combat the adverse effects of climate change on food security and agriculture sustainability. The Post-Green Revolution high-yielding, biotic stress-resistant but drought-susceptible varieties were bred for the targeted irrigated ecosystem. Exploitation of genetic variation, use of holistic modified breeding strategies, and direct selection for grain yield (Kumar et al., 2014) combining traits contributing yield advantage has been suggested as an appropriate approach to develop drought tolerant rice cultivars. Several studies involving grain yield as the main selection criterion have identified stable, consistent and large-effect QTLs for grain yield under reproductive-stage drought stress (Bernier et al., 2007; Venuprasad et al., 2009; Vikram et al., 2011; Ghimire et al., 2012; Mishra et al., 2013; Swamy and Kumar, 2013; Yadaw et al., 2013; Dixit et al., 2014a,b). Some of these identified QTLs have been deployed to develop high-yielding reproductive-stage drought-tolerant rice varieties (Kumar et al., 2014). However, although the reproductive stage is the growth stage at which rice yield is most affected by drought, rice yields in farmers' fields may be reduced by drought occurring at any growth stage. Therefore, continued efforts are needed to identify QTLs and traits that confer improved rice yields under multiple types of drought stress.

Phenotypic screening involving direct selection for grain yield under reproductive-stage drought stress, non-stress (control), multiple environments (upland and lowland), locations, seasons/years has led to the identification of major and consistent effect grain yield QTLs (Kumar et al., 2014; Dixit et al., 2015; Sandhu et al., 2015). Identification of co-located genetic regions associated with grain yield under drought, root and seedling establishment traits have opened up further possibilities for improving rice yield under drought stress.

To date, most of the reports identifying major-effect QTLs for rice yield under drought have involved selection of a traditional variety as the drought tolerance donor. Many traditional upland/aus cultivars showed a high level of drought tolerance, but their drought tolerance is often linked to undesirable traits, such as low yield potential and/or tall plant height (Vikram et al., 2015). Therefore, preliminary studies confirming the efficiency of the drought donor in a breeding program is necessary (Kumar et al., 2014). One such potential donor is the traditional variety Dular, which was identified in early rice genotype screening efforts as a drought-tolerant cultivar that maintained grain yield under reduced water availability and showed deep root growth (De Datta et al., 1975). Subsequent reports highlighted Dular as a traditional variety with the ability to maintain seminal root elongation under drought, but with less lateral root formation and plasticity in response to fluctuating soil moisture (Bañoc et al., 2000), and as one of the most deep-rooted genotypes of the diverse OryzaSNP panel in solution culture, lysimeter, and in upland and lowland drought field studies (Henry et al., 2011; Gowda et al., 2012; Shrestha et al., 2013; Wade et al., 2015). Given the previous research identifying Dular as deep-rooting, we hypothesized that RILs with the highest grain yield under drought would be those with highest root growth at depth, which would be related to seedling stage crown root formation.

To harness the drought tolerance (yield and root growth) traits of Dular for use in breeding for multiple drought stress environments, this study involved recombinant inbred lines (RILs) derived from a cross of IR64-21 with Dular. The aim was to identify the genetic regions for grain yield, yield-related agronomic and root development traits under direct seeded upland and transplanted lowland reproductive-stage drought stress and non-stress conditions.

## Materials and methods

Recombinant inbred lines (RILs–F_7_) were developed at the T.T. Chang Genetic Resources Center at the International Rice Research Institute (IRRI), Los Baños, Philippines, using single-seed descent from F_2_ progenies of the cross between IR64-21 (single plant selection from IR64, IRGC 117268) and Dular (purified line, IRGC117266). Dular is an upland adapted, drought-tolerant traditional cultivar from India (McNally et al., 2009) and has showed consistent performance in drought screening at IRRI. Dular is early maturing and has low-yield potential due to low tillering. IR64-21 is a lowland adapted, semi dwarf, medium-duration, high-yielding cultivar with good grain quality that has been widely grown across large areas of Asia and Africa. Eight field and two greenhouse experiments were conducted at IRRI (14°11′ N 121°15′ E, 21 m above sea level) during the dry seasons (DS) of 2013 and 2014 (Table 1). The soil type in the experimental fields is a Maahas clay loam; isohyperthermic mixed typic tropudalf (Zhao et al., 2006; Venuprasad et al., 2009).

**Table 1:** Details of the experiments conducted for the phenotyping of rice IR64-21 × Dular recombinant inbred lines mapping population.

**Experiment number**	**Experiment type**	**Year/Environment/Season/Treatment**	**Experimental design**	**Pop size**	**Rep**	**Seeding date**
1	Field study	2013DS_UNS	49 × 10 AL	490	2	Dec. 22, 2012
2	Field study	2013DS_URS	49 × 10 AL	490	2	Jan. 21, 2013
3	Field study	2013DS_LNS	49 × 10 AL	490	2	Dec. 22, 2012
4	Field study	2013DS_LRS	49 × 10 AL	490	2	Dec. 21, 2012
5	Field study	2014DS_UNS	49 × 10 AL	490	2	Jan. 03, 2014
6	Field study	2014DS_URS	49 × 10 AL	490	2	Jan. 03, 2014
7	Field study	2014DS_LNS	30 × 10 AL	304	2	Dec. 13, 2013
8	Field study	2014DS_LRS	30 × 10 AL	304	2	Dec. 13, 2013
9	Basket study	2013WS_GH	RCBD	300	3	July 10, 2013
10	Lysimeter study	2015WS_GH	RCBD	302	3	July 31, 2015

*DS, dry season; WS, wet season; UNS, upland non-stress; URS, upland reproductive-stage drought stress; LNS, lowland non-stress; LRS, lowland reproductive stage drought stress; GH, greenhouse; AL, alpha lattice; RCBD, randomized complete block design; Pop Size, Population size; Rep, number of replicates*.

### Management of upland and lowland field experiments

In this study, upland non-stress (UNS) refers to experiments in non-flooded, non-puddled, rainfed, naturally well drained fields where seeding was done when dry, whereas lowland non-stress (LNS) refers to experiments under flooded, puddled, transplanted and anaerobic conditions. Upland drought stress (URS) and lowland drought stress (LRS) refer to experiments comprising cyclic naturally imposed drought stress at the reproductive stage.

Upland experiments were conducted during the dry seasons of 2013 and 2014 with 480 F_7_ RILs in α- lattice design with two replications of 2-m single-row plots with row spacing of 25 cm, except in the 2014 DS UNS experiment in which 1.5-m single row plots were used. The two parents, IR64-21 and Dular, were used as checks and were replicated five times within each replication. Seeds were dry direct-seeded in aerobic soil using a seeding rate of 5 g per row. Fertilizer with the proportion 100N:40P:40K was applied; P and K were applied as basal doses at 10 days after seeding (DAS) and N was applied in three equal splits at 10, 25, and 45 DAS. Hand weeding was done as needed and insecticide (Furadan; carbofuran, 1 kg a.i ha^−1^) was used to control insect pests when necessary. The stress experiments were sprinkler-irrigated twice weekly during establishment and early vegetative growth. Tensiometers were installed at a depth of 30 cm to record the soil water tension. Drought stress was initiated at 35 DAS and plots were irrigated only when most lines were wilted and exhibited severe leaf drying and the soil water potential fell below −50 kPa. This cyclic reproductive stage drought stress allows effective reproductive-stage drought screening for broad range growth duration genotypes (Lafitte et al., 2004). Upland non-stress experiments received the same cultural practices as the stress experiments except that the irrigation was continued twice weekly up to 1 week before harvest. The total rainfall accumulated during stress period in the upland experiments (35–90 DAS) was 54.6 mm and 28.4 mm during 2013DS and 2014DS, respectively.

The same 480 RILs were used in the lowland experiment conducted during 2013DS while 300 randomly selected RILs were used in the 2014DS lowland experiments. Soil in the 2014DS field study was characterized as having a water-extractable pH of 6.5, 33.3 mg kg^−1^ P (Olsen), 441 mg kg^−1^ K, 40% clay, 21% sand, and 39% silt. Seeds were sown in a raised bed nursery; 21-day-old seedlings were transplanted to the main field. In the 2013DS lowland experiments, one seedling per hill was planted in an α- lattice design with two replications of 5 m single-row plots, with hill and row spacing of 0.2 m. In the 2014DS lowland experiments, three or four seedlings per hill were planted in an α- lattice design with two replications in plots of seven 1-m rows, with hill spacing of 0.2 m and row spacing of 0.25 m. After transplanting, ~5 cm of standing water was maintained in the lowland fields before the initiation of drought stress at 50 DAS in both 2013DS and 2014DS. Plots in the drought stress treatment were rewatered when the soil water potential dropped to −50 kPa. The total rainfall accumulated during the stress period in the lowland (50–100 DAS) was 197.4 and 14.2 mm during 2013DS and 2014DS, respectively. During the 2014DS LRS experiment, water potential dropped below−50 kPa twice (68 and 91 DAS) and was rewatered twice (79 and 102 DAS). Irrigation was maintained up to 10 days before harvest for the non-stress experiments. Furadan (carbofuran, 1 kg a.i ha^−1^) was applied to control stem borer and other insects when necessary.

### Characterization of seedling stage, root, and agronomic traits

#### Field studies

Days to emergence was recorded in the 2014DS upland stress and lowland experiments. Seedling stage growth rate, in terms of the increase in height in centimeters per week from 28 to 56 DAS, was recorded in both stress and non-stress upland experiments of 2014DS.

Destructive sampling of three plants (shoot and root crown) was done at 42 DAS in the 2014DS upland stress experiment to evaluate crown root traits. Shoots were separated from the roots and then dried and weighed to determine shoot dry mass while the roots were stored in 50% ethanol for root counting. Numbers of crown (nodal) roots, adventitious (mesocotyl-borne) roots and seminal roots were counted manually and the sum of all axial crown root types was defined as the total root number. The mesocotyl length was measured using a centimeter scale. All crown root number and mesocotyl length values from the three root samples per plot were averaged.

In the 2014DS lowland experiments, root samples were acquired using a 4-cm diameter core sampler (fabricated at IRRI, Los Baños, Philippines) to a depth of 60 cm. One core per plot was sampled at 104–109 DAS, soil cores were divided into 15-cm segments and roots were washed by repeatedly mixing the soil with water in a container and pouring the root-water suspension over a 1-mm plastic sieve. Roots were scanned (Calibrated Color Optical Scanner STD4800, Epson) and analyzed (WinRHIZO Pro v. 2013e, Regent Instruments) to determine root length density in each depth segment. Sampled roots were dried and weighed to measure the root dry weight. Xylem sap bleeding rate, defined as the weight of xylem sap bled after detopping normalized by the shoot mass of each hill, was measured at 89 or 90 DAS as described by Morita and Jun (2002). Shoots of one hill per plot in the drought stress experiment were cut about 10 cm above the soil surface. Sap exuded from the cut stems was collected with a preweighed cotton towel wrapped in plastic. Canopy temperature was measured on six dates between 71 and 97 DAS by the tractor-based phenotyping system (Tanger et al., 2017).

In all field studies, days to 50% flowering, plant height (cm) at maturity and grain yield (kg ha^−1^) were recorded (Table 2). Days to 50% flowering (DTF) was recorded as the number of days from seeding to the day on which 50% of the panicles had emerged. Specifically, it is at growth stage 5 (inflorescence emergence), code 55 of the BBCH-scale for rice (Lancashire et al., 1991). The height of three randomly chosen plants per plot was measured at maturity from ground level to the tip of the highest panicle and then averaged. At physiological maturity, grains were harvested from each plot, dried (moisture content ~14%) and weighed to calculate grain yield in kg ha^−1^. Shoot samples were collected at harvest from the 2014DS UNS, LNS, and LRS experiments, oven dried, threshed and weighed for the calculation of total biomass (kg ha^−1^) and harvest index (HI). HI was calculated as the ratio of grain weight to the total above-ground plant weight. Total numbers of panicles and tillers were counted from the whole plant samples and number of tillers and panicles per square meter were calculated from the 2014DS UNS.

**Table 2:** Details of traits measured for the phenotyping of IR64-21 × Dular recombinant inbred mapping population.

**Year/Environment/Season/Treatment**	**Trait name**
2013DS_UNS	DTF, PHT, GY
2013DS_URS	DTF, PHT, GY, LR
2013DS_LNS	DTF, PHT, GY
2013DS_LRS	DTF, PHT, GY
2014DS_UNS	DTF, PHT, GY, BIO, EVV, GR, HI, PAN, TIL
2014DS_URS	DTF, PHT, GY, 6PYT, 6SDW, EME, GR, LR, ARN, CRN, SRN, RAS, TRN, ML
2014DS_LNS	DTF, PHT, GY, BIO, HI
2014DS_LRS	DTF, PHT, GY, EME, HI, LR, DR, LAT, RBN, RDW, RL, BIO, BR, CT
2013DS_GH	PHT, TIL, SDW, DR, SR, RL, RDW, TRN
2014WS_GH	PHT, TIL, SDW, PU, TWU, WUE, PDW, RD, RDW

*DS, dry season; WS, wet season; UNS, upland non-stress; URS, upland reproductive-stage drought stress; LNS, lowland non-stress; LRS, lowland reproductive stage drought stress; GH, greenhouse; DTF, days to 50% flowering; PHT, plant height (cm); GY, grain yield (kg ha^-1^); LR, leaf rolling score; BIO, biomass (kg ha^-1^); EVV, early vegetative vigor (score); GR (cm/week). HI, harvest index; PAN, number of panicles; TIL, number of tillers; 6PYT, plant height at 6 weeks after seeding (cm); 6SDW, shoot dry weight at 6 weeks after seeding (kg ha^-1^); EME, days to emergence; ARN, number of adventitious roots; CRN, number of crown roots; SRN, number of seminal roots; RAS, number of root axial sites; TRN, total number of roots; ML, mesocotyl length (mm); DR, percent deep roots; SR, percent shallow roots; LAT, percent lateral roots; RBN, root branching number; RDW, root dry weight (g); RLD, root length density (cm cm^-3^); BR, bleeding rate (sap/g); CT, canopy temperature; PU, phosphorus uptake (mg P/tiller); TWU, total water uptake (L); WUE, water use efficiency (g/L); PDW, panicle dry weight with rachis (g); RD, maximum root depth (cm)*.

#### Greenhouse basket root experiment

In 2013WS, seedling-stage root growth of 300 RILs (the same RILs used in 2014DS lowland experiments) was measured using the basket method as described by Uga (2012). Open stainless-steel mesh baskets (top diameter of 7.5 cm, depth of 5.0 cm and mesh size of 2 mm) were filled with sieved, dried soil from the IRRI farm. Each soil-filled basket was placed on a support made of PVC pipe (7.5-cm diameter, 15-cm height) and arranged in plastic trays (56 × 36.5 cm, 15-cm height; 28 baskets per tray). Each basket included a ring indicator dividing the basket into the upper and lower halves. Roots emerging above the ring indicator (shallow roots) represented an angle of from 0 to 50°, while roots emerging below the ring indicator (deep roots) represented an angle of from 50 to 90° with respect to the horizontal soil surface centered on the plant stem. The 300 RILs were grown in three replicates, with three successive germination dates for the three replicates (Jul 8, 2013, Aug 9 2013 and Sep 13 2013). Seeds were germinated in Petri dishes lined with filter paper, then transferred to the middle soil surface of each basket after 2–6 days. The nutrient solutions were monitored and recorded daily. The set-up was watered using tap water for the first week, half strength Yoshida solution for the second week, and full-strength Yoshida solution from the third week onward. Average temperature and humidity were 30°C and 60%, 30°C and 50% and 28.5°C and 45% in replicates 1, 2, and 3, respectively. The plants were harvested from 26 to 29 days after planting. Plant height, tiller number, root length, total number of roots and percent deep and shallow roots were recorded. Percent deep roots and shallow roots were computed as follows:

$$\begin{array}{l} \%\;deep\;roots=\left(\frac{number\;of\;deep\;roots}{total\;root\;number\;}\right)\times 100\%\\ \%\;shallow\;roots=\left(\frac{number\;of\;shallow\;roots}{total\;root\;number\;}\right)\times 100\% \end{array}$$

Percent deep roots were used as a proxy for root growth angle (Uga, 2012). The shoots and roots were dried for 3 days in an oven at 60°C and the root dry weight and shoot dry weight were recorded.

#### Greenhouse lysimeter study

A greenhouse lysimeter study was conducted in 2015WS to evaluate deep root growth, water uptake and phosphorus uptake under drought in the IR64-21 × Dular population. Seeds of IR64-21, Dular and the same 300 RILs that were evaluated in the 2014DS lowland field experiment were germinated for 4 days in Petri dishes and then three plants were transplanted into each lysimeter. The lysimeters consisted of PVC cylinders (height: 95 cm, diameter: 20 cm) lined with a plastic sheet and filled with 23 kg of sieved, dry upland soil to a height of 70 cm, with an additional 20 cm of lowland paddy soil added on top of the upland soil. Representative soil samples were sent to the IRRI Analytical Services Laboratory for analysis of Kjeldahl N (0.13%), available P (49.67 mg kg^−1^), Exchangeable K (2.26 meq 100 g^−1^), Mg (7.63 meq 100 g^−1^), Ca (14.87 meq 100 g^−1^), pH (6.1), clay (35.7%), sand (17%), and silt (47.3%). Each genotype was planted in three replicates in a randomized complete block design within the greenhouse and each replicate was arranged within a concrete tank within the greenhouse. Five grams of complete fertilizer (14-14-14 N-P-K) were added to each lysimeter before planting. At 10 days after planting, the plants were thinned to one seedling per lysimeter. Each lysimeter was then sealed at the top with a plastic sheet wrapped around the base of the plant to prevent water loss due to evaporation from the soil surface so that transpiration could be assessed gravimetrically. The drought stress treatment was initiated by removing the rubber stoppers from three holes at the bottom of each lysimeter to drain them at 29, 32, and 34 days after germination (DAG) in replicates 1, 2 and 3, respectively. Weekly weighing of the lysimeters and simultaneous imaging of shoots were initiated at 31, 33 and 35 DAG in replicates 1, 2, and 3, respectively using a mechanical hoist and camera suspended from a gantry crane as described by Kijoji et al. (2012) to determine water uptake and apparent leaf area. During the course of the study, temperature in the greenhouse averaged 33.35°C; relative humidity averaged 62.74% and mid-day light levels (10–14 h) averaged 602.13 μmol m^−2^ s^−1^. Days to flag leaf appearance and to flowering were recorded for each lysimeter. At 58 DAG, one tiller was sampled from each lysimeter to be used for phosphorus-uptake measurements. Phosphorus uptake per tiller was determined on dried, ground tissue spectrophotometrically using the method described by Murphy and Riley (1962). Harvest of each lysimeter was initiated at 85 DAG based on observations of maturity for each plant. At the time of harvest, the number of tillers was counted, plant height was measured and the shoot was cut to determine shoot dry weight (SDW) and panicle dry weight. The soil was removed from each lysimeter by pulling out the plastic liner sheet and the maximum root depth was determined. The soil segment below the depth of 60 cm was sampled and roots were carefully washed from the soil and dried to determine root dry weight (RDW) >60 cm. Total water uptake (TWU) was determined based on the difference between the initial and final weights of each lysimeter and water use efficiency (WUE) was calculated as SDW/TWU. Weekly water uptake rates were normalized by the apparent leaf area to determine normalized water uptake rates in each lysimeter.

### Statistical analysis of phenotypic data

Analysis of variance (ANOVA) was conducted using PBTools V 1.4.0. (PBTools, 2014). Mixed model analysis was conducted to calculate the trait means, which were later used for further analysis. The model used for ANOVA for alpha lattice design was:

$$\begin{array}{l} P_{ijk}=M+R_{i}+B_{j}\left(R_{i}\right)+L_{k}+e_{ijk} \end{array}$$

Where, P_ijk_ is the measurement recorded on a plot, M is the mean over all plots and R, B, L and e refer to replications, blocks, lines and error, respectively. Data of yield experiments for computation of means, heritability and least square difference (LSD) were analyzed using PBtools 1.4.0 taking the effect of replications and block within replications. Broad sense heritability was calculated as:

$$\begin{array}{l} H=\frac{\sigma^{2}G}{\sigma^{2}G+\frac{\sigma^{2}E}{r}} \end{array}$$

where H is the broad sense heritability of the experiment, σ^2^G is the genetic variance, σ^2^E is the error variance and r is the number of replications in the experiment. Correlation among traits was calculated using STAR V 2.0.1 (STAR, 2014) and was visualized graphically through multidimensional scaling (MDS) using the same program. Briefly, the MDS model accounts for each object or event as a point in a multidimensional space wherein the points are arranged within the space; therefore, the distances between pairs of points have the best fit relation to the similarities among pairs of objects (Wilkinson, 1996).

Multi-environment analysis of the 480 genotypes under eight field experiments was performed using the AMMI-1 (Additive Main Effect and Multiplicative Interaction) stability model in PBTools v.1.4.0. The AMMI model equation according to Gauch and Zobel (1996) for T genotypes and S environments was:

$$\begin{array}{l} \;Y_{ij}=\mu +g_{i}+e_{j}+\sum_{n-1}^{n^{\prime }}\lambda_{n}\alpha_{in}\gamma_{jn}+\theta_{ij}\\ \theta_{ij}\sim N\left(0,\sigma^{2}\right);i=1,2,\ldots ,T;j=1,2,\ldots ,S. \end{array}$$

where *Y*_*ij*_ is the mean yield of the *i*^*th*^ genotype in the *j*^*th*^ environment; μ is the grand mean; *g*_*i*_ is the *i*^*th*^ genotypic effect; *e*_*j*_ is the *j*^*th*^ environment effect; λ_*n*_ is the eigen value of the PCA axis *n*; α_*in*_ and γ_*jn*_ are the *i*^*th*^ genotype *j*^*th*^ environment PCA scores for the PCA axis *n*; θ_*ij*_ is the residual; *n'* is the number of PCA axis retained in the model.

To relate seedling-stage roots traits, reproductive-stage root traits, water uptake under drought and yield, a path analysis was conducted using *lavaan* script in R v. 3.3 using the subset of 304 IR64-21 × Dular RILs. Parameters measured in multiple experiments or on multiple dates were averaged, including days to emergence, total root number, biomass at harvest, canopy temperature and yield under drought stress.

### Genetic analysis

#### Leaf collection, DNA extraction, PCR and PAGE

The genotyping work was carried out at IRRI's Genotyping Services Lab (GSL). Eight fresh leaves from one replication of each genotype in 2014DS UNS were collected in bulk and immediately kept on ice. The leaves were collected in glassine bags with proper labeling. A portion of these leaves was placed in individual tubes, ground and stored at −20°C for later processing. The DNA from all 480 genotypes and the two parents were extracted using a modified CTAB protocol developed by Murray and Thompson (1980). The DNA was dissolved in 200 μL of TE (Tris-EDTA) buffer. DNA solutions were stored at −20°C. Polymerase chain reaction (PCR) was performed using 15-μL reactions. Amplifications were performed using 40 ng of DNA, 1 × PCR buffer, 100 μM dNTPs, 250 μM oligonucleotide primers and 1 unit of *Taq* polymerase. This mix was prepared in 96-well polycarbonate plates and the thermocycling procedure followed the method described by Panaud et al. (1996). Amplified products were resolved using high-resolution 8% (v/v) polyacrylamide gel electrophoresis (PAGE) (CBS scientific, model MGV-202–33) as described by Sambrook et al. (1989). The gel was run in a 1xTBE buffer at 95 volts for 1–3 h, depending on the SSR marker product size. DNA fragments were stained with SYBER Safe™ and visualized using a UV trans-illuminator.

#### Parental survey for polymorphism and whole population genotyping

A total of 400 rice SSR markers from available rice genetic sequence maps were used to perform a polymorphic survey between IR64-21 and Dular. The details of the primers such as marker position, chromosome position, expected product size and annealing temperature were taken from Gramene Genome Browser (www.gramene.org). PCR, PAGE and gel documentation of the images were performed as described earlier. The bands were scored as either 1 or 2 based on their banding pattern. A total of 115 polymorphic markers spread evenly across the genome were selected and used for generating the genotyping data of the whole population including the parents. Primers designated to each marker were used in PCR and the subsequent PAGE and gel documentation were performed as described earlier.

#### Linkage map construction and QTL analysis

The entry means of all phenotypic traits were correlated with the genotypic data of the respective lines using two software programs; QTL Network 2.1 (Yang et al., 2008) and Windows QTL Cartographer version 2.5 (Wang et al., 2012). Two software programs were used due to the different analysis tools available in each program and also to identify consistent and stable QTLs identified by both programs. A genetic locus was confirmed only if consistent with both programs. The QTL Network software identifies putative regions within the QTLs based on one-dimensional genome scan considering chosen candidate intervals as cofactors. A mixed linear model framework was used to perform the mapping procedure. F-statistics based on Henderson method III were used for hypothesis testing. The critical F-value was calculated based on 1,000 permutation tests to minimize the genome-wide type I error. A window size of 1 and a walk speed of 1.0 cM were used for the whole genome scan. A significant level of *p* < 0.001 was maintained for QTL detection in the whole experiment. The linkage map was constructed and analysis was then repeated using the QTL cartographer software (Wang et al., 2012). Composite interval mapping (CIM) with default parameters (300 permutation time, 5% of significance level, model 6; standard model, method 3; forward and backward method with walk speed 2 cM) was performed; LOD explained by each QTL was calculated. The flanking markers intervals and positions, peak marker and position (P) and additive effect (A) of the QTLs was calculated using QTL network and also confirmed with Windows QTL Cartographer version 2.5; the logarithm of the odds (LOD) score of the QTLs was calculated using Windows QTL Cartographer version 2.5 and h^2^ (heritability) using QTL network 2.1. As the absolute additive effect changes with severity of stress, the additive effect was presented as a percentage of the population mean as: %A (percent additive effect) = A (additive effect) × 100/Population mean.

The single QTL (*qDTY*_*1.1*_*, qDTY*_*1.3*_*, qDTY*_*8.1*_), two QTL combination (*qDTY*_*1.1*_+*qDTY*_*1.3*_*, qDTY*_*1.1*_+*qDTY*_*8.1*_, *qDTY*_*1.3*_+*qDTY*_*8.1*_) and three QTL combination (*qDTY*_*1.1*_+*qDTY*_*1.3*_+*qDTY*_*8.1*_) represents the allelic profile of contributing QTL parent allele in the respective QTL regions in particular genotype.

#### Identification of candidate genes and other colocating QTLs

Functionally characterized genes within the indentified QTLs as well as previously identified QTLs colocating with the identified QTLs in this study were determined using Q-TARO (QTL Annotation Rice Online, http://qtaro.abr.affrc.go.jp/cgi-bin/gbrowse) database based on the physical position of both primers flanking the QTL (Yamamoto et al., 2012). The chromosome number and the genomic region were entered in the genome browse panel using the format Chr#:genome start.genome end. For example, Chr1:8,000,000.10,000,000 was entered to indicate that the physical position 8,000,000–10,000,000 bp in chromosome 1 was the region inquired for candidate gene and other colocating QTL identification.

## Results

### Yield and related trait characterization

The mean performance of the 480 genotypes and the checks (Dular and IR64-21) is presented in Table 3. Dular consistently out-yielded IR64-21 by 21–99% in all experiments except for the 2014 LNS experiment wherein IR64-21 out-yielded Dular by 94%. Recorded grain yields of the RIL population for URS experiments ranged from 0 to 947.4 kg ha^−1^ and 90.4–4,844.3 kg ha^−1^ for the UNS experiments. For the lowland experiments, grain yield ranged from 2.2–4,607.8 kg ha^−1^ under drought stress and 81.5–6,578.7 kg ha^−1^ under non-stress. A number of genotypes out-yielded both checks in all experiments except for the URS experiments wherein Dular showed the highest grain yield. Dular flowered earlier and had taller plant height at maturity compared to IR64-21 and RIL in all experiments. The mean days to 50% flowering (DTF) and plant height at maturity of the population were intermediate to both parents.

**Table 3:** Mean, range, LSD, Pr > F and heritability for grain yield, days to 50% flowering and plant height at maturity observed for Dular, IR64-21 and the RIL population under rainfed lowland and upland conditions.

**Trait (unit)**	**Y/S/E/T**	**Dular**	**IR64-21**	**Population mean**	**Population range**	**LSD_*0.5*_**	**Pr > F**	**H**
Days to 50% flowering	2013DS_UNS	64	87	79	64–108	8	0.000	59
	2014DS_UNS	72	99	85	71–102	6	0.000	82
	2013DS_URS	84	97	94	78–118	13	0.000	46
	2014DS_URS	86	84	96	81–121	16	0.003	18
	2013DS_LNS	79	88	85	67–103	6	0.000	89
	2014DS_LNS	77	83	85	69–96	5	0.000	82
	2013DS_LRS	74	87	82	65–106	5	0.000	94
	2014DS_LRS	77	97	87	66–169	15	0.000	100
Plant height at maturity (cm)	2013DS_UNS	101.82	64.22	95.64	48.6–138.2	17.7	0.000	89
	2014DS_UNS	91.98	69.94	97.47	58.2–136.4	16.4	0.000	88
	2013DS_URS	68.49	39.22	60.04	28.2–91.5	14.3	0.000	86
	2014DS_URS	73.84	44.56	66.40	36.4–95.5	14.1	0.000	84
	2013DS_LNS	96.81	61.08	87.50	45.2–120.6	16.9	0.000	89
	2014DS_LNS	114.37	85.66	115.70	58.0–166.4	16.3	0.000	96
	2013DS_LRS	98.08	83.58	122.47	62.8–348.0	78.9	0.000	45
	2014DS_LRS	69.40	44.90	75.01	39.4–207.5	37.8	0.000	58
Grain yield (kg ha^−1^)	2013DS_UNS	2,857.60	643.33	1,867.44	90.4–4,844.3	1,577.2	0.000	56
	2014DS_UNS	4,205.92	1,783.22	2,404.55	255.6–4,828.6	1,665.0	0.000	54
	2013DS_URS	364.16	5.02	13.82	0.0–140.9	91.6	0.000	0
	2014DS_URS	1,720.71	18.19	111.96	0.7–947.4	294.6	0.000	50
	2013DS_LNS	2,673.72	2,104.22	2,413.33	81.5–6,578.7	1,727.0	0.000	72
	2014DS_LNS	1,707.94	3,313.62	2,286.91	401.6–5,950.7	1,249.7	0.000	68
	2013DS_LRS	2,242.72	312.80	927.08	2.2–2,921.4	678.2	0.000	87
	2014DS_LRS	551.66	140.07	729.04	50.9–4,607.8	833.6	0.000	44

*Y, Year; S, season; E, ecosystem; T, treatment; DS, dry season; UNS, upland non-stress; URS, upland reproductive-stage drought stress; LNS, lowland non-stress; LRS, lowland reproductive-stage drought stress; LSD_0.5_, least significant difference at 0.5% level of significance; H, broad sense heritability*.

### Effect of drought stress on yield and related traits

Under upland reproductive-stage drought stress conditions, the average grain yield reduction (GYR) of Dular and IR64-21 was observed to be 73 and 99%, respectively, while the RIL population exhibited a GYR of 71-100%. A GYR of 42 and 90% were observed for Dular and IR64-21, respectively, under lowland reproductive-stage drought while a GYR of 0–99% was observed in the RIL population (Table 4). Under upland stress conditions, DTF of the RIL population was delayed by 1–36 days while it was only delayed by 0–9 days under lowland stress conditions. Plant height of the RIL population was reduced by 4–67 cm in upland stress and 9–78 cm in lowland stress compared to non-stress conditions. The biomass and harvest index of Dular decreased by 32 and 48%, respectively, while it decreased by 50 and 78%, respectively, in IR64-21. A reduction of 4–84 and 0–87% to the biomass and harvest index, respectively, was observed in the RIL population under reproductive-stage drought stress compared to non-stress.

**Table 4:** Yield reduction, delay in flowering, plant height reduction, biomass reduction and harvest index reduction observed for Dular, IR64-21 and the RIL population under drought stress in rainfed lowland and rainfed upland conditions compared to their non-stress counterparts.

**Criterion**	**Y/S/E**	**Dular**	**IR64-21**	**Population range**
Grain yield reduction (%)	2013DS Upland	87	99	87.4–100
	2014DS Upland	60	99	54.1–100
	2013DS Lowland	16	85	0.0–100
	2014DS Lowland	68	95	1.9–95.3
Days to flowering reduction	2013DS Upland	20	10	1–37
	2014DS Upland	14	−14	0–34
	2013DS Lowland	−5	−1	0–18
	2014DS Lowland	1	11	0–9
Plant height at maturity reduction (cm)	2013DS Upland	33.3	25.0	5–67
	2014DS Upland	18.1	25.4	4–67
	2013DS Lowland	17.6	24.6	1–75
	2014DS Lowland	28.7	38.7	18–82
Biomass reduction (%)	2014DS Lowland	32	50	3.8–84.2
Harvest index reduction (%)	2014DS Lowland	48	78	0–86.9

*Y, Year; S, season; E, ecosystem; T, treatment; DS, dry season*.

### Correlation among traits

**Figure 1:**
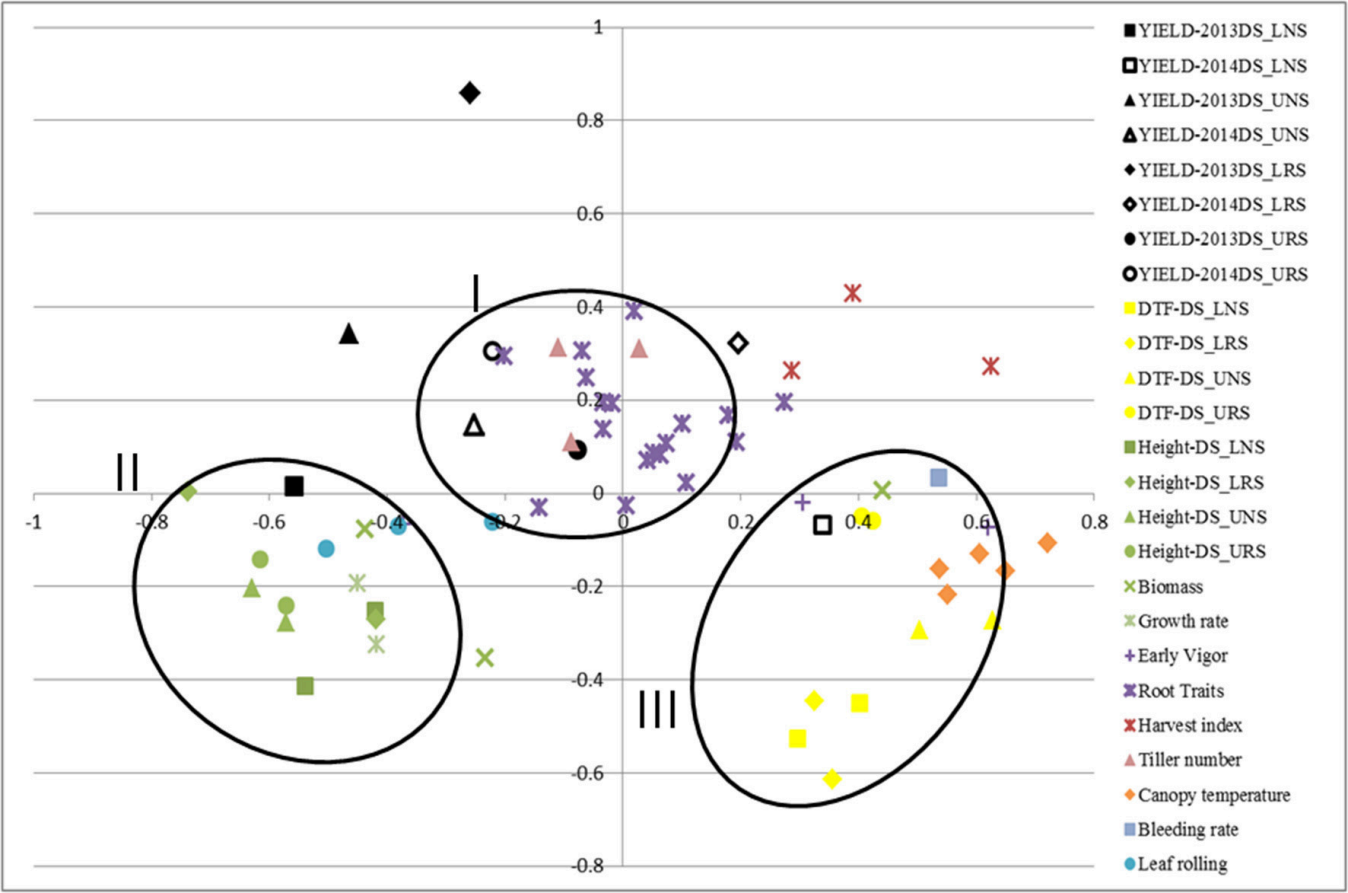
Multidimensional scaling of traits showing the correlation of rice traits related to yield and tolerance to drought for IR64-21 × Dular recombinant inbred lines under rainfed lowland and upland conditions. DTF, days to 50% flowering; DS, dry season; UNS, upland non-stress; URS, upland reproductive-stage drought stress; LNS, lowland non-stress; LRS, lowland reproductive-stage drought stress.

Correlations among traits were calculated using STAR V 2.0.1 and were visualized graphically through MDS using the same program (Figure 1). Formation of clusters indicates good correlation among the clustered traits. The closeness of the same trait measured in different environments indicates less effect of the environment on these traits. MDS analysis showed the clustering of traits; grain yield, root traits, harvest index and number of tillers in one cluster (I) while plant height, biomass, growth rate and leaf rolling were in another cluster (II). A third cluster (III) was formed by DTF, canopy temperature, bleeding rate and early vigor.

**Figure 2:**
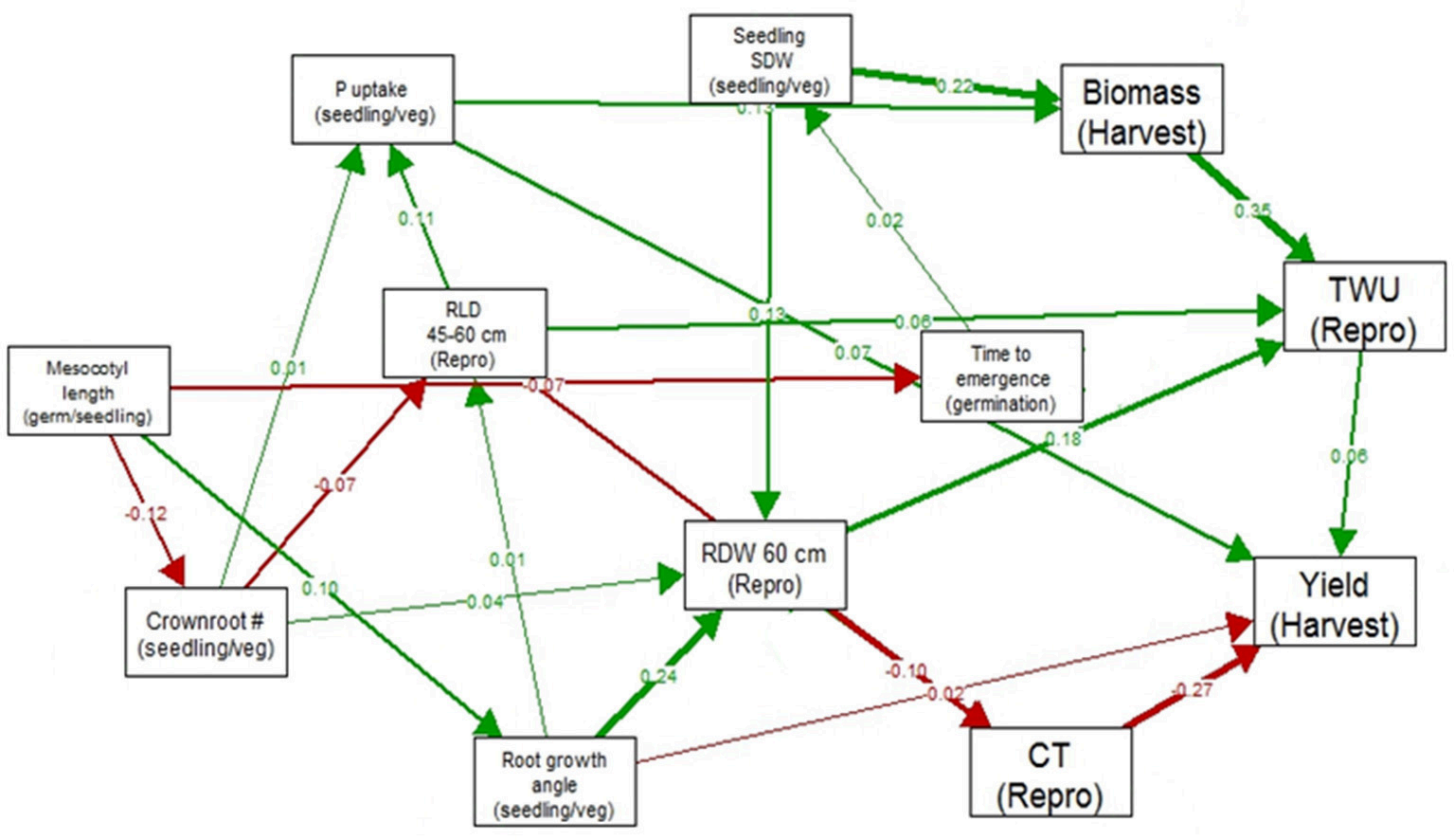
Path analysis relating traits measured at germination stage, seedling/vegetative stage, reproductive stage and harvest in 300 IR64-21 × Dular RILs. Values shown are correlation coefficients (green for positive and red for negative), and the width of the arrow represents the strength of the relationship.

In the path analysis relating traits measured at germination stage, seedling/vegetative stage, reproductive stage and harvest, correlation coefficients ranged below 0.36 (Figure 2). The strongest correlations between growth stages were between total water uptake and biomass at harvest, canopy temperature and yield, seedling stage shoot dry weight and root dry weight below 60 cm, and root growth angle and root dry weight below 60 cm. The strongest paths linking seedling stage to harvest were via (a) seedling stage shoot dry weight which was related to reproductive-stage biomass and total water uptake, which were related to yield under drought, and (b) root growth angle, which was related to root dry weight below 60 cm, which affected total water uptake and was related to yield under drought.

### Identified QTLs for grain yield and other traits

**Figure 3:**
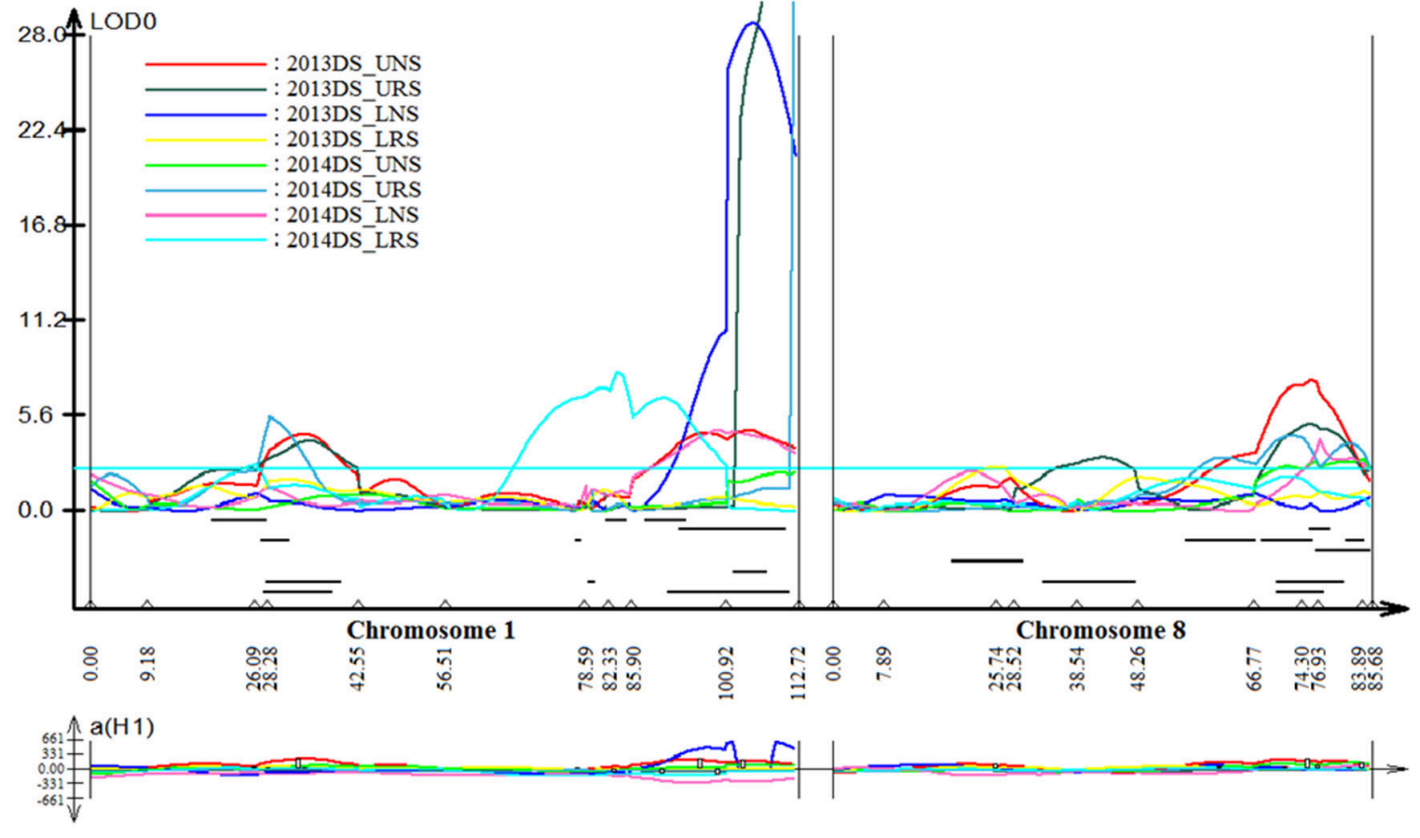
QTL likelihood curve of LOD score for grain yield QTLs identified across environments and seasons on chromosomes 1 and 8 using QTL cartographer version 2.5.

**Figure 4:**
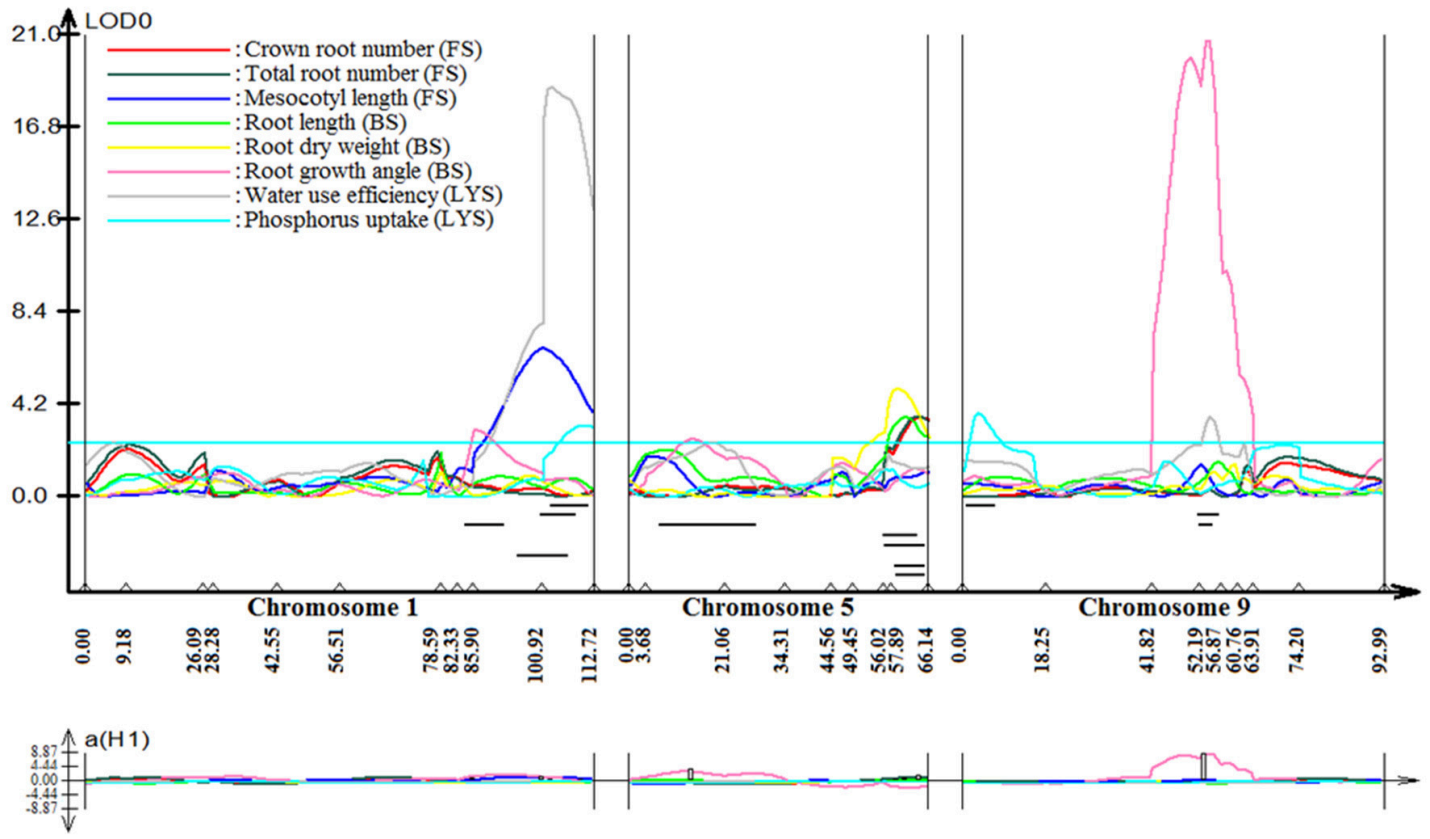
QTL likelihood curve of LOD score for root traits QTLs identified on chromosomes 1, 5, 8, and 9 using QTL cartographer version 2.5. FS, Field study (2014DS_URS); BS, basket study; LYS, lysimeter study. LOD graph generated.

Stable, consistent-effect QTLs with significant *p-*values were identified; three for grain yield on chromosomes 1 and 8 (Figure 3) and four for root traits on chromosomes 1, 5, 8, and 9 (Figure 4). A hotspot genetic locus was identified as *qDTY*_*1.1*_ on chromosome 1 where multiple QTLs for grain yield, plant height (*qPHT*_*1.1*_), biomass (*qBIO*_*1.1*_), growth rate, leaf rolling (*qLR*_*1.1*_), mesocotyl length (*qRT*_*1.1*_), bleeding rate, water-use efficiency (*qWUE*_*1.1*_) (Table 5) and phosphorus uptake (*qPU*_*1.1*_) were detected across years and environments between markers RM11943 and RM3482 (Figure 4). The novel grain yield QTLs, *qDTY*_*1.3*_ and *qDTY*_*8.1*_ with heritability values ranging from 3.1–5.1 and 4.4–7.6, respectively, and additive effects of 9.0–43.7% and 11.6–39.7%, respectively were identified. The grain yield QTL *qDTY*_*1.3*_ on chromosome 1 between RM292 and RM11013 was consistent under both non-stress and reproductive stage drought stress under upland conditions. The grain yield QTL *qDTY*_*8.1*_ on chromosome 8 between markers RM80 and RM230 showed consistent effects under both nonstress and reproductive-stage drought stress, *qRT*_*9.1*_ on chromosome 9 between RM434 and RM257 was identified for root growth angle (Figure 4). QTL *qRT*_*9.1*_ showed a heritability of 29%, an additive effect of 16.7 and 15.4% for % deep roots and % shallow roots, respectively. The percent deep root QTL was contributed by Dular, whereas the % shallow root QTL was contributed by IR64-21 (Table 5). A genetic region of 9.9 cM on chromosome 5 was associated with root traits including root length and root dry weight measured in the basket study and crown root number at the seedling stage measured in 2014DS URS (Figure 4) with heritability of 5.8, 7.9, and 3.5%, respectively (Table 5).

**Table 5:** QTLs identified in the IR64-21 × Dular RILs mapping population for grain yield, grain yield attributing traits and root development traits under rainfed lowland and upland conditions.

**QTL**	**Trait name**	**Year/Environment/Season/Treatment**	**Interval**	**P(cM)**	**Range (cM)**	**%A**	***h*^2^**
*qDTY_*1.3*_*	GY	2014DS_LRS	RM292-RM583	26.1	17.2–28.1	10.85	3.1
	GY	2014DS_URS	RM292-RM583	28.1	22.2–33.3	34.60	5.1
	GY	2013DS_UNS	RM583-RM11013	34.3	29.3–41.3	8.95	4.1
	GY	2013DS_ URS	RM583-RM11013	34.3	28.3–40.3	43.68	3.8
*qBIO_*1.1*_*	Biomass	2014DS_UNS	RM11943-RM3482	104.9	100.9–107.9	9.50	12.0
	Biomass	2014DS_LNS	RM11943-RM3482	105.9	100.9–110.9	9.35	10.7
	Biomass	2014DS_LRS	RM11943-RM3482	105.9	101.9–109.9	9.50	13.5
*qDTY_*1.1*_*	GY	2013DS_LNS	RM11943-RM3482	105.9	103.9–108.9	29.85	23.5
	GY	2013DS_UNS	RM11943-RM3482	106.9	100.9–111.9	11.18	4.6
	GR	2014DS_UNS	RM11943-RM3482	104.9	102.9–106.9	19.97	35.3
*qPHT_*1.1*_*	PHT	2014DS_LRS	RM11943-RM3482	104.9	101.9–107.9	17.30	22.9
	PHT	2013DS_UNS	RM11943-RM3482	104.9	103.9–106.9	17.48	49.2
	PHT	2014DS_UNS	RM11943-RM3482	104.9	102.9–106.9	15.19	51.9
	PHT	2013DS_URS	RM11943-RM3482	104.9	102.9–106.9	17.23	36.1
	PHT	2014DS_URS	RM11943-RM3482	104.9	102.9–106.9	16.24	46.3
	PHT	2013DS_LNS	RM11943-RM3482	105.9	103.9–107.9	20.80	49.7
	PHT	2013DS_LRS	RM11943-RM3482	105.9	103.9–107.9	16.95	45.4
	PHT	2014DS_LNS	RM11943-RM3482	109.9	105.9–111.9	14.30	18.9
*qLR_*1.1*_*	LR	2013DS_URS	RM11943-RM3482	104.9	101.9–106.9	27.66	29.1
	LR	2014DS_URS	RM11943-RM3482	104.9	102.9–106.9	30.13	31.1
*qRT_*1.1*_*	ML	2014DS_URS	RM11943-RM3482	104.9	94.9–110.9	6.88	5.1
*qWUE_*1.1*_*	WUE	2015WS_GH	RM11943-RM3482	105.9	102.9–109.9	6.84	23.1
*qDTY_*8.1*_*	GY	2014DS_URS	RM80-RM419	72.8	68.8–76.3	34.17	4.4
	GY	2013DS_UNS	RM419-RM230	75.3	70.8–76.9	11.60	7.6
	GY	2013DS_URS	RM419-RM230	75.3	70.8–81.9	39.71	5.5
*qRT_*9.1*_*	DR	2013WS_GH	RM434-RM257	53.2	50.8–55.2	16.76	28.9
	SR	2013WS_GH	RM434-RM257	53.2	50.8–55.2	−15.37	28.9
*qRT_*5.1*_*	RL	2013WS_GH	RM87-RM334	58.9	56.0–63.9	3.55	5.8
	RDW	2013WS_GH	RM87-RM334	59.9	56.0–64.9	8.89	7.0
	CRN	2014DS_URS	RM87-RM334	63.9	58.9–65.9	4.50	3.5

*GY, grain yield; GR, growth rate; PHT, plant height at maturity; LR, leaf rolling; ML, mesocotyl length; DR, % deep roots; SR, % shallow roots; RL, root length; RDW, root dry weight; CRN, crown root number; WEU, water use efficiency; PU, phosphorus uptake; DS, dry season; UNS, upland non-stress; URS, upland reproductive-stage drought stress; LNS, lowland non-stress; LRS, lowland reproductive-stage drought stress; GH, green house experiment; P, position of the peak of the QTL; %A, additive effect of the QTL as percent of the population mean*.

### Stability of grain yield under multiple environments

Stable genotypes across different environments were identified among the 480 genotypes using the AMMI-1 stability model. The 480 genotypes were analyzed and ranked based on mean grain yield across eight environments. Twelve high-yielding genotypes with PC1 scores of −4.0 to 4.0 (indicating lower sensitivity with different growing environments) with different QTL combinations were selected together with IR64-21 and Dular (parents) and plotted based on the mean grain yield and PC1 score in order to get a clear picture of stable genotypes across all environments (Supplementary Figure 1). The RILs IR 92132-366-1-1-1-1-1-1 with QTL combination *qDTY*_*1.1*_+*qDTY*_*8.1*_, IR 92132-119-1-1-1-1-1-1; *qDTY*_*1.3*_+*qDTY*_*8.1*_, IR 92132-2035-1-1-1-1-1-1; *qDTY*_*1.1*_+*qDTY*_*1.3*_+*qDTY*_*8.1*_, and IR 92132-1826-1-1-1-1-1-1; *qDTY*_*1.3*_+*qDTY*_*8.1*_ were identified as stable genotypes that stood out for grain yield across environments as well as a number of root traits (Table 6).

**Table 6:** Characterization of stable genotypes across different environments.

**RIL**	**QTL class**	**Grain yield**						**Root traits**					
		**UNS**	**URS**	**LNS**	**LRS**	CRN^*^	TRN^*^	ML^*^	RL^*^	RDW^*^	**DR**	**SR**	**PU**
IR 92132-1937-1-1-1-1-1-1	1	4,004	398	2,435	3,23	34	35	13.7	20.5	0.63	53.8	46.2	1.46
IR 92132-2033-1-1-1-1-1-1	1	3,982	461	4,631	3,75	30	30	20.8	23.1	0.53	63.5	36.5	2.35
IR 92132-2035-1-1-1-1-1-1	1	3,082	138	1,841	1,297	28	29	18.9	23.7	0.65	52.2	47.8	1.81
IR 92132-366-1-1-1-1-1-1	2	3,439	348	1,185	1,395	33	34	18.9	24.2	0.73	45.0	55.0	1.10
IR 92132-1271-1-1-1-1-1-1	2	2,254	419	1,877	2,55	46	46	16.7	22.2	0.74	46.9	53.1	2.21
IR 92132-1826-1-1-1-1-1-1	3	3,506	516	2,908	1,210	35	36	23.1	23.2	0.60	55.4	44.6	2.02
IR 92132-1926-1-1-1-1-1-1	3	2,660	791	1,712	1,256	44	46	15.8	21.4	0.44	50.4	49.6	2.34
IR 92132-613-1-1-1-1-1-1	4	4,090	771	2,870	1,013	31	32	23.8	18.0	0.44	61.8	38.2	0.90
IR 92132-750-1-1-1-1-1-1	4	2,070	670	3,876	1,711	43	44	19.5	18.8	0.44	62.6	37.4	2.52
IR 92132-232-1-1-1-1-1-1	5	3,970	43	1,956	1,360	39	39	27.8	16.8	0.38	60.7	39.3	1.42
IR 92132-621-1-1-1-1-1-1	5	3,562	171	2,713	2,161	35	36	14.9	20.1	0.53	58.2	41.9	1.45
IR64-21	6	1,783	18	2,709	2,26	35	36	15.9	25.8	0.28	44.9	55.1	1.36
WPM		2,405	112	2,350	8,28	32	33	18.1	21.5	0.58	47.7	52.3	1.63

*DS, dry season; UNS, upland non-stress; URS, upland reproductive-stage drought stress; LNS, lowland non-stress; LRS, lowland reproductive-stage drought stress; CRN, crown root number; TRN, total root number; ML, mesocotyl length; RL, root length; RDW, root dry weight; DR, % deep roots; SR, % shallow roots; WUE, water use efficiency; PU, phosphorus uptake; WPM, whole population mean. QTL class, 1, qDTY_1.1_ + qDTY_1.3_ + qDTY_8.1;_ 2, qDTY_1.1_ + qDTY_8.1_; 3, qDTY_1.3_ + qDTY_8.1_; 4, qDTY_1.1_ + qDTY_1.3_; 5, qDTY_1.1_; 6, no QTL*.

* *CRN, TRN, ML, RL, and RDW were measured at seedling stage during 2014DS URS*.

### Physiological response of selected genotypes

**Figure 5:**
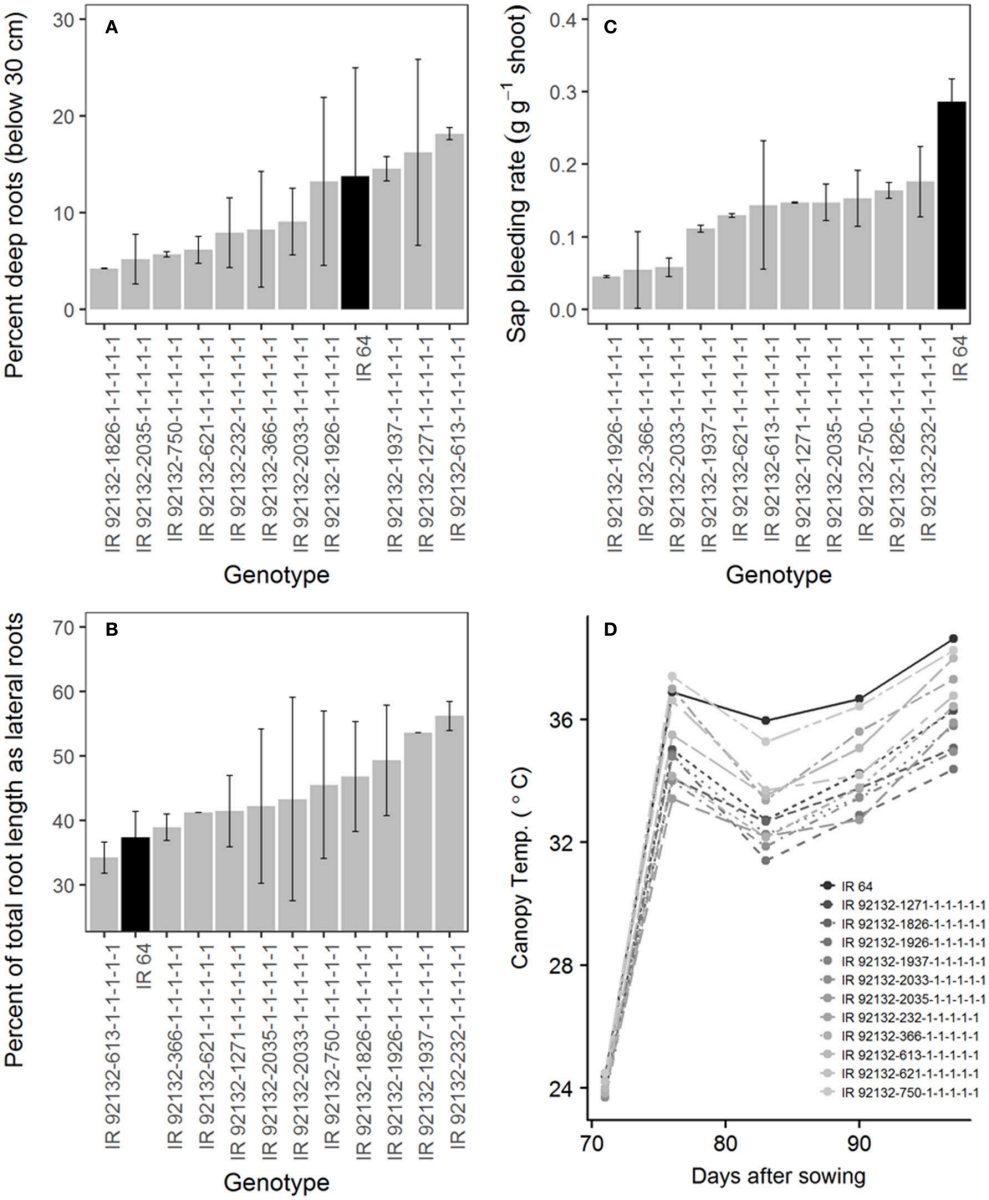
Field drought experiment (2014DS_LRS) **(A,B)** Root growth; **(C)** sap bleeding rate; **(D)** canopy temperature of the most stable, high-yielding genotypes of the IR64-21 × Dular population.

In comparison with IR64-21 in the greenhouse lysimeter experiment, the most stable and highest yielding IR64-21 × Dular RILs (Table 6) showed generally higher root dry weight at depth (Supplementary Figure 2A) but similar maximum root depth (Supplementary Figure 2B) and higher WUE (Supplementary Figure 2C) but similar water uptake rates (Supplementary Figure 2D). In the field under drought, the most stable and highest yielding IR64-21 × Dular RILs showed higher percentages of the total root length as lateral roots (Figure 5B), lower sap bleeding rates (Figure 5C) and lower canopy temperatures (Figure 5D) than IR64-21, but similar percent deep roots (Figure 5A).

### Effect of identified QTLs on grain yield

Compared to genotypes without *qDTY*_*1.1*_, the genotypes with *qDTY*_*1.1*_ showed grain yield improvement (GYI) of 2.9% in 2013DS and 55% in 2014DS under URS (Table 7), reflecting the higher severity of stress in 2013DS, in which observed yield reductions of the population ranged up to 99% (Table 4). The genotypes with *qDTY*_*1.1*_ showed GYI of 70.8 and 1.6% under lowland non-stress and reproductive-stage drought stress, respectively (Table 7). The genotypes with *qDTY*_*1.3*_, however, showed higher GYI in 2013 upland with 108.9%, than in 2014 upland with 24.1% GYI. Similar results were observed under lowland conditions (Table 7). Grain yield improvements of genotypes with *qDTY*_*8.1*_ over the lines without *qDTY*_*8.1*_ during 2013 and 2014, were 97.4 and 74%, respectively, in upland and 3.0 and 16.2%, respectively, in lowland conditions (Table 7). The QTL combination *qDTY*_*1.1*_ + *qDTY*_*8.1*_ showed the highest GYI, followed by *qDTY*_*1.3*_+*qDTY*_*8.1*_ and *qDTY*_*1.1*_+*qDTY*_*8.1*_+*qDTY*_*1.3*_ across upland/lowland reproductive-stage drought stress. *qDTY*_*1.3*_ and *qDTY*_*8.1*_, individually and combined, showed a high GYI even under severe drought stress, while *qDTY*_*1.1*_, alone, resulted in high GYI under non-stress and medium stress but not under severe upland stress.

**Table 7:** Percentage grain yield improvement of genotypes possessing QTL (+ QTL) over lines not possessing QTL (– QTL) for grain yield under reproductive stage drought stress.

**QTL**	**Year/Environment/Season/Treatment**	**+ QTL (mean value)**	**− QTL (mean value)**	**%GYI (– QTL)**	**IR64-21**	**%GYI (IR64-21)**
*qDTY_*1.1*_*	2013DS_UNS	2, 015.8	1, 660.8	21.4	643.3	213.3
	2014DS_UNS	2, 503.9	2, 222.5	12.7	1, 783.2	40.4
	2014DS_URS	134.1	86.5	55.0	18.2	637.3
	2013DS_LNS	2871.6	1, 681.4	70.8	2, 104.2	36.5
	2013DS_LRS	951.2	936.1	1.6	312.8	204.1
*qDTY_*8.1*_*	2013DS_UNS	2, 109.4	1, 664.2	26.8	643.3	227.9
	2014DS_UNS	2, 509.9	2, 255.9	11.3	1783.2	40.8
	2013DS_URS	18.4	9.3	97.4	5.0	267.7
	2014DS_URS	141.9	81.6	74.0	18.2	680.3
	2013DS_LNS	2, 474.2	2, 298.3	7.7	2, 104.2	17.6
	2014DS_LNS	2, 357.2	2, 154.4	9.4	3, 313.6	−
	2013DS_LRS	950.6	923.1	3.0	312.8	203.9
	2014DS_LRS	764.3	657.9	16.2	140.1	445.6
*qDTY_*1.3*_*	2013DS_UNS	2, 032.3	1, 643.0	23.7	643.3	215.9
	2014DS_UNS	2, 494.9	2, 302.0	8.4	1, 783.2	39.9
	2013DS_URS	18.9	9.0	108.9	5.0	276.7
	2014DS_URS	123.9	99.8	24.1	18.2	581.2
	2014DS_LNS	2, 290.6	2, 243.8	2.1	3, 313.6	−
	2013DS_LRS	922.8	752.0	22.7	312.8	195.0
	2014DS_LRS	748.6	6, 94.3	7.8	140.1	434.5
*qDTY_*1.1*_* + *qDTY_*8.1*_*	2013DS_UNS	2, 287.5	1, 583.1	44.5	643.3	255.6
	2014DS_UNS	2, 639.3	2, 258.6	16.9	1, 783.2	48.0
	2013DS_URS	17.3	10.8	60.2	5.0	244.0
	2014DS_URS	165.7	73.4	125.8	18.2	810.9
	2013DS_LNS	2, 980.7	1, 971.6	51.2	2, 104.2	41.7
	2013DS_LRS	1, 058.3	932.4	13.5	312.8	238.3
*qDTY_*1.3*_* + *qDTY_*8.1*_*	2013DS_UNS	2, 440.3	1, 688.2	44.5	643.3	279.3
	2014DS_UNS	2, 685.7	2, 321.5	15.7	1, 783.2	50.6
	2013DS_URS	24.3	9.1	167.4	5.0	383.9
	2014DS_URS	146.1	85.6	70.8	18.2	703.6
	2013DS_LNS	2, 528.1	2, 425.5	4.2	2, 104.2	20.1
	2014DS_LNS	2, 378.3	2196.7	8.3	3, 313.6	−
	2013DS_LRS	1, 016.3	923.3	10.1	312.8	224.9
	2014DS_LRS	832.3	678.2	22.7	140.1	494.2
*qDTY_*1.1*_* + *qDTY_*1.3*_*	2013DS_UNS	2, 339.5	1, 714.1	36.5	643.3	263.7
	2014DS_UNS	2, 601.2	2, 277.7	14.2	1, 783.2	45.9
	2013DS_URS	16.4	11.9	37.1	5.0	226.1
	2014DS_URS	155.1	90.2	72.0	18.2	753.1
	2013DS_LNS	2, 784.8	1, 991.6	39.8	2, 104.2	32.3
	2013DS_LRS	942.7	897.2	5.1	312.8	201.4
*qDTY_*1.1*_* + *qDTY_*1.3*_* + *qDTY_*8.1*_*	2013DS_UNS	2, 860.9	1, 620.5	76.5	643.3	344.7
	2014DS_UNS	2, 779.7	2, 268.7	22.5	1, 783.2	55.9
	2013DS_URS	16.4	9.8	67.2	5.0	226.3
	2014DS_URS	170.1	75.2	126.2	18.2	835.1
	2013DS_LNS	3, 143.0	2, 011.7	56.2	2, 104.2	49.4
	2014DS_LNS	2, 453.3	2, 343.9	4.7	3, 313.6	−
	2013DS_LRS	1, 107.8	951.1	16.5	312.8	254.2
	2014DS_LRS	769.0	725.5	6.0	140.1	449.0

*DS, dry season; UNS, upland non-stress; URS, upland reproductive-stage drought stress; LNS, lowland non-stress; LRS, lowland reproductive-stage drought stress*.

## Discussion

With the root and drought response traits measured in this study from a large population (>300 RILs) at times ranging from seedling stage to harvest, we aimed to (1) integrate the relationships among traits at different growth stages in terms of their effects on yield and (2) identify the genetic loci controlling the traits that have an effect on yield under drought. We hypothesized that, although traits measured at seedling and vegetative stage are chronologically distant from the harvest; some key intermediate traits measured over the growing seasons could link seedling-stage traits with grain yield under drought. Given the wide differences between IR64-21 and Dular in terms of their phenotype and their response to reproductive-stage drought stress, we had the opportunity to explore and observe which traits have an effect on yield under drought and which combinations of QTLs were most effective under drought in the RIL population.

Delayed flowering, decreased plant height and reduced grain yield are some of the effects of drought stress (Lafitte et al., 2004; Zhao et al., 2010; Vikram et al., 2011; Kumar et al., 2014). The reduction in grain yield upon imposition of stress on the populations was 71–100% in the upland experiments and 0–99% in the lowland experiments indicating the severity of stress (Table 4). Different levels of stress among experiments are always desirable to eliminate the effect of yield potential and to select better drought-resistant germplasm (Bernier et al., 2007). Under reproductive-stage drought stress, Dular outperformed IR64-21 supporting the suitability of breeding efforts involving Dular as a source of genetic loci that enhance grain yield under drought stress.

Clustering of root traits with grain yield in the MDS analysis signifies the role of root traits in maintaining water or nutrient uptake under reproductive-stage drought stress conditions in this population. According to the path analysis, the strongest links between early- and late-season traits were from seeding stage shoot dry weight or seedling stage root growth angle (Figure 2). The identification of a relationship between seedling- and vegetative-stage root traits from the greenhouse experiments (root growth angle measured in the basket experiment and root dry weight below 60 cm in the lysimeter experiment) rather than from the field may have been due to the different root growth components measured in the two types of environments (crown root numbers vs. angles) as well as the high degree of variability inherent to field root studies.

This study identified QTL *qRT*_*9.1*_ for root growth angle, which is adjacent to the previously reported QTL *qGY*_*9.1*_, and *qGY*_*9.2*_ for grain yield and *qEVV*_*9.1*_ for early vegetative vigor in the Aus276/3^*^IR64 population under direct-seeded conditions (Sandhu et al., 2015), for root length (Price et al., 1999, 2002; Uga et al., 2010; Sandhu et al., 2013), root thickness (Steele et al., 2006, 2013), and root number (Li et al., 2005). Furthermore, meta analysis from a 675-root QTL database from 12 populations (Courtois et al., 2009) showed the presence of root architecture-related QTLs colocated or adjacent to *qRT*_*9.1*_. Although the effect of the identified genetic regions requires a proper validation in different genetic backgrounds and environments, under the present climate change scenario, successful introgression following marker-assisted breeding may be used to improve the root system architecture of the widely cultivable popular rice varieties. The contribution of deep- and shallow-root enhancing alleles by Dular and IR64-21, respectively, signifies the role of both parents in maintaining the nutrient-water balance in the promising lines, as IR64-21 is a lowland-adapted parent with shallower root growth and Dular is rainfed-adapted parent with a deeper root system.

The genomic locus *qDTY*_*1.1*_ stood out as a hotspot for grain yield, agronomic and physiological traits. The major, consistent and large effect of *qDTY*_*1.1*_ on grain yield under non-stress, reproductive-stage drought stress, and direct seeded conditions has been reported previously in different backgrounds, Swarna (with additive effect of 13%), IR64 (with additive effect of 8, 11, 15, and 24%) and MTU1010 (using different donors such as N22, Dhagaddeshi, Aus276 and Kali Aus (Vikram et al., 2011; Sandhu et al., 2014, 2015, 2016). This region has also been observed to be associated with plastic responses to drought stress, including enhancing deep root growth and shoot growth regulation (Vikram et al., 2015; Wade et al., 2015). *qDTY*_*8.1*_ from this study was observed to be located adjacent to *qGY*_*8.1*_, identified in an Aus276/3^*^IR64 population under direct-seeded conditions, validating the effect of drought-tolerant loci in the IR64 background across variable environmental and cultivation conditions. Similarly, the genetic locus *qRT*_*5.1*_ (with additive effect of 3.6, 8.9, and 4.5%), associated with root traits on chromosome 5 in the present study, was located in the same region previously associated with QTLs for nutrient uptake (with additive effect of 12 and 23%) and root traits (with additive effect of 8 and 6%) under direct-seeded conditions (Sandhu et al., 2015) in Aus276/3^*^IR64 population. This indicates the possibility of presence of conserved allelic region in different donors and help to harness the potential of multiple donors enhancing water-nutrient uptake under stress conditions. This colocation signifies the relationship of root traits with nutrient uptake in improving grain yield under drought and direct-seeded conditions. The identification of *qDTY*_*1.1*_, *qDTY*_*8.1*_, *qRT*_*5.1*_, and *qRT*_*9.1*_ in earlier studies clearly demonstrates the stability of these QTLs across different genetic backgrounds and the identification of a novel QTL, *qDTY*_*1.3*_, presents an alternate resource for introgression of drought tolerance into susceptible varieties.

In terms of QTL combinations, *qDTY*_*1.3*_+*qDTY*_*8.1*_, *qDTY*_*1.1*_+*qDTY*_*8.1*_ and *qDTY*_*1.1*_+*qDTY*_*8.1*_+*qDTY*_*1.3*_ showed the highest GYI under severe stress, indicating the positive interaction of *qDTY*_*1.1*_, *qDTY*_*8.1*_, *qDTY*_*1.3*_, and *qDTY*_*8.1*_. Of the individual QTLs, *qDTY*_*1.3*_ individually showed a GYI under severe drought stress. The GYI from all the QTLs identified was attributed to the Dular allele, as indicated by the additive effect of the QTLs (Table 5). The grain yield advantage of promising lines with the identified QTLs individually and in combination over that of IR64-21 was significant. The positive interactions between loci *qDTY*_*1.1*_+*qDTY*_*8.1*_ and *qDTY*_*1.3*_+*qDTY*_*8.1*_ might be useful in further QTL pyramiding and marker-assisted QTL introgression programs. The promising lines with *qDTY*_*1.1*_+*qDTY*_*8.1*_ and *qDTY*_*1.3*_+*qDTY*_*8.1*_ also showed grain yield stability across variable growing environments (upland, lowland, drought stress and non-stress; Table 6), further supporting the existing positive interactions among the identified loci. RIL IR 92132-366-1-1-1-1-1-1 with *qDTY*_*1.1*_+*qDTY*_*8.1*_ was one of the most ideal genotypes with higher root dry weight compared to IR64-21 and Dular (Table 6). However, IR 92132-1271-1-1-1-1-1-1 having *qDTY*_*1.1*_+*qDTY*_*8.1*_, showed shorter mesocotyls and lower WUE (Table 6, Supplementary Figure 2), as well as lower yield and less stability compared to IR 92132-366-1-1-1-1-1-1. This genotypic variation among lines with the same QTLs indicates the importance of QTL x QTL interactions in combination with root architecture characteristics that affect nutrient and water uptake as well as seedling establishment. Similarly, RILs IR 92132-119-1-1-1-1-1-1, IR 92132-1826-1-1-1-1-1-1 and IR 92132-1926-1-1-1-1-1-1 all showed the presence of *qDTY*_*1.3*_+*qDTY*_*8.1*_, however, the stability and mean grain yield of IR 92132-119-1-1-1-1-1-1 and IR 92132-1826-1-1-1-1-1-1 were better than IR 92132-1926-1-1-1-1-1-1 across environments. The proper balance between root traits, seedling establishment traits and yield attributing traits may have played a role in the differences in grain yield among selected RILs.

The better performance of yield-stable genotypes across variable growing environments appears to be largely attributed by drought tolerance-related root traits (Table 6). Longer mesocotyls were expected to result in earlier time to emergence and a greater number of crown roots at the seedling stage, however, only the latter was confirmed in this study. The observation that the most stable and highest yielding IR64-21 × Dular RILs differed from IR64-21 in some (particularly % lateral roots) but not all deep-rooting traits (such as maximum root depth) suggests that the differences in root architecture may be more related to lateral root growth rather than nodal root elongation. Furthermore, the differences in traits related to water uptake between IR64-21 and the most stable and highest yielding IR64-21 × Dular RILs indicate that the drought response of these lines is conferred by a combination of root architecture and root functional traits. This observation is in agreement with previous reports that Dular contrasts with IR64-21 in terms of root hydraulic parameters under drought, including xylem cell diameter, root hydraulic conductivity, sap bleeding rate and aquaporin expression (Henry et al., 2011). These results are also supported by the MDS analysis in which root traits clustered with grain yield under drought stress.

Previously identified genes and other QTLs in the QTL regions identified in the study may give insights as to why these QTLs confer grain yield enhancement and stability under drought stress conditions (Supplementary Tables 1, 2). Three functionally characterized genes were identified within *qDTY*_*1.3*_, namely S-Adenosyl-l-methionine synthetase3 (*OsSAMS3*), S-Adenosyl-l-methionine synthetase2 (*OsSAMS2*) and photoassimilate defective1 (*phd1*) (Li C. et al., 2011; Li W. et al., 2011). *OsSAMS3* and *OsSAMS2* affect fertility, germination rate and flowering time in rice while phd1 affects photosynthetic activity and biomass and grain production. QTLs identified in this study colocate with previously identified QTLs associated with root and grain yield-related agronomic traits such as root branching index and number of filled grains per panicle (Zhuang et al., 2001; Li et al., 2005; Horii et al., 2006) (Supplementary Figure 3). Within the QTL region *qDTY*_*8.1*_ on chromosome 8 identified in the present study, the gene rice authentic His-containing phosphotransfer 1, associated with lateral and crown root development, osmotic adjustment and seed setting under drought, was identified in knockdown experiments (Sun et al., 2014). Other genes, such as *OsMADS7* that controls floral organ formation in rice and proton gradient regulation 5 that controls the plant's photosynthetic capacity (Cui et al., 2010; Nishikawa et al., 2012), were also identified to be within *qDTY*_*8.1*_. Drought tolerance genes, such as dehydration-responsive element-binding transcription factor 1G, heat shock factor class B 2b (Chen et al., 2008; Xiang et al., 2013) and QTLs for root-to-shoot ratio, maximum root length, relative germination vigor and filled grain weight per plant, have also been reported within *qDTY*_*8.1*_ (Zhuang et al., 1997; Price et al., 2002; You et al., 2006).

## Conclusion

The identified genetic regions associated with grain yield, yield attributing and root development traits under reproductive-stage drought stress and the identified promising lines in this study may facilitate future marker-assisted QTL pyramiding/introgression breeding programs to improve rice yield under drought conditions. Fine mapping, precise introgression of genetic regions with positive interactions and evaluation of consistent performance across environments and genetic backgrounds may further improve grain yield and may provide insight into the physiological and molecular characterization of genetic loci.

## Availability of data and materials

The data sets supporting the results of this article are included within the article.

## Author contributions

MC was involved in conducting the experiment, recording observations and drafting the article; NS and SD was involved in experimental analysis, interpretation of data and revising the manuscript; NAAS was involved in conducting the experiment and recording observations; MN and KM developed the RILs; AH was involved in designing the greenhouse experiment and the critical revision of the manuscript; MD and KM were involved in revision of the manuscript; and AK conceived the study and contributed to the critical revision of the manuscript. All authors approved the final version of the manuscript.

### Conflict of interest statement

The authors declare that the research was conducted in the absence of any commercial or financial relationships that could be construed as a potential conflict of interest.
