# Supplementary material for: Genetic Loci Governing Grain Yield and Root Development under Variable Rice Cultivation Conditions
Source: Front Plant Sci. 2017 Oct 16;8:1763. doi: 10.3389/fpls.2017.01763 (PMC5650699; doi:10.3389/fpls.2017.01763)
Supplement: Supplementary file 1 [file Table1.DOCX]

Supplementary Material

**Exploiting genetic loci enhancing grain yield and root development under variable cultivation conditions**

Margaret Catolos^1,2^, Nitika Sandhu^1^, Shalabh Dixit^1^, Noraziyah Abd Aziz Shamsudin^1,3^, Elizabeth Naredo^1^, Kenneth McNally^1^, Amelia Henry^1^, Ma. Genaleen Diaz^2^ and Arvind Kumar^1^*

*** Correspondence:** Arvind Kumar: a.kumar@irri.org

# Supplementary Tables

**Supplementary TABLE 1| Functionally characterized genes within the indentified QTLs in IR64** $\boldsymbol{\times}$ **Dular RILs mapping population for rice traits related to yield and drought tolerance under rainfed lowland and upland conditions determined using Q-TARO.**

| Chromosome region | Gene | Objective character | | Genome start-end | | Method of Isolation | | Reference | |  |
| --- | --- | --- | --- | --- | --- | --- | --- | --- | --- | --- |
| *qDTY_1.3_*  (Chr1:  9-18Mbp) | *S-Adenosyl-l-methionine synthetase3* | | Dwarfism,  Fertility,  Germination rate,  Flowering time | | 10705422-10708091 | | Knockdown | | Li et al., 2011a | |
| *qDTY_1.3_*  (Chr1:  9-18Mbp) | *S-Adenosyl-l-methionine synthetase2* | | Dwarfism,  Fertility,  Germination rate,  Flowering time | | 12506499-12508973 | | Knockdown | | Li et al., 2011a | |
| *qDTY_1.3_*  (Chr1:  9-18Mbp) | *Photoassimilate defective1* | | Chlorophyll content,  Photosynthetic activity,  Biomass,  Grain production. | | 15224863-15229762 | | Mutant | | Li et al., 2011b | |
| *qDTY_8.1_*  (Chr8:  25-35Mbp) | *Rice authentic His-containing phosphotransfer 1* | | Root development,  enhanced lateral root growth,  gravitropism,  Leaf senescence by cytokinin-mediated inhibition of chlorophyll content,  Tiller numbers,  Seed setting rate,  Dwarfism with reduced internode lengths,  Osmotic tolerance with enhanced lateral roots and crown roots under mannitol treatment | | 27993412-27997515 | | Knockdown | | Sun et al., 2014 | |
| *qDTY_8.1_*  (Chr8:  25-35Mbp) | *OsMADS7* | Floral organ formation. | | 26595185-26600266 | | Knockdown | | Cui et al., 2010 | |  |
| *qDTY_8.1_*  (Chr8:  25-35Mbp) | *Proton gradient regulation 5* | Photosynthetic capacity. | | 28465839-28466926 | | Knockdown | | Nishikawa et al., 2012 | |  |
| *qDTY_8.1_*  (Chr8:  25-35Mbp) | *Dehydration-Responsive Element Binding transcription factor1G* | Drought tolerance. | | 27408597-27415080 | | Overexpression | | Chen et al., 2008 | |  |
| *qDTY_8.1_*  (Chr8:  25-35Mbp) | *Heat shock factor class B 2b* | Drought and salinity tolerance. | | 27468770-27478343 | | Knockdown Overexpression | | Xiang et al., 2013 | |  |
