# Supplementary material for: Genetic Loci Governing Grain Yield and Root Development under Variable Rice Cultivation Conditions
Source: Front Plant Sci. 2017 Oct 16;8:1763. doi: 10.3389/fpls.2017.01763 (PMC5650699; doi:10.3389/fpls.2017.01763)
Supplement: Supplementary file 2 [file Table2.DOCX]

Supplementary Material

**Exploiting genetic loci enhancing grain yield and root development under variable cultivation conditions**

Margaret Catolos^1,2^, Nitika Sandhu^1^, Shalabh Dixit^1^, Noraziyah Abd Aziz Shamsudin^1,3^, Elizabeth Naredo^1^, Kenneth McNally^1^, Amelia Henry^1^, Ma. Genaleen Diaz^2^ and Arvind Kumar^1^*

*** Correspondence:** Arvind Kumar: a.kumar@irri.org

**Supplementary TABLE 2| QTLs of other traits co-locating with the identified QTLs in IR64** $\boldsymbol{\times}$ **Dular RILs mapping population for rice traits related to yield and drought tolerance under rainfed lowland and upland conditions determined using the genome browser of Q-TARO.**

| Chromosome region | QTL | Character | Marker used | QTL start-end | Population used | Parent A/B | Direction | Reference |
| --- | --- | --- | --- | --- | --- | --- | --- | --- |
| *qDTY_1.3_*  (Chr1:  9-18Mbp) |  | Root Branching index | SSR | 10753550-26104011 | BC_1_F_5_ | Akihikari/ IRAT109 | A | Horii et al., 2006 |
| *qDTY_1.3_*  (Chr1:  9-18Mbp) |  | Number of filled grains per panicle | RFLP | 5759741-39646396 | RIL | Zhenshan 97B/ Milyang 46 |  | Zhuang et al., 2001 |
| *qDTY_1.3_*  (Chr1:  9-18Mbp) | *rfw1b* | Drought tolerance | RFLP | 7476658-35715948 | DHL | IRAT109/ Yuefu | A | Li et al., 2001 |
| *qDTY_8.1_*  (Chr8:  25-35Mbp) | *8_4* | Root to shoot ratio | RFLP | 24274675-27825769 | RIL | Bala/ Azucena |  | Price et al., 2002 |
| *qDTY_8.1_*  (Chr8:  25-35Mbp) | *8_4* | Maximum root length | RFLP | 24274675-27825769 | RIL | Bala/ Azucena |  | Price et al., 2002 |
| *qDTY_8.1_*  (Chr8:  25-35Mbp) | *qRGV-8* | Relative germination vigor(RGV) | SSR | 26582935-28166213 | RIL | Zhenshan 97/ IRAT109 | B | Jun et al., 2006 |
| *qDTY_8.1_*  (Chr8:  25-35Mbp) |  | filled grain weight per plant (GYD) | RFLP | 25681306-27825769 | F_2_ and F_3_ | Tesanai/ CB | A | Zhuang et al., 1997 |
