# Supplementary material for: Genetic Loci Governing Grain Yield and Root Development under Variable Rice Cultivation Conditions
Source: Front Plant Sci. 2017 Oct 16;8:1763. doi: 10.3389/fpls.2017.01763 (PMC5650699; doi:10.3389/fpls.2017.01763)
Supplement: Supplementary file 3 [file Image1.PDF]

Supplementary Material

Exploiting genetic loci enhancing grain yield and root development under variable cultivation conditions

Margaret Catolos[1,2], Nitika Sandhu[1], Shalabh Dixit[1], Noraziyah Abd Aziz Shamsudin[1,3], Elizabeth Naredo[1], Kenneth McNally[1], Amelia Henry[1], Ma. Genaleen Diaz[2] and Arvind Kumar[1]*

*** Correspondence:** Arvind Kumar: a.kumar@irri.org

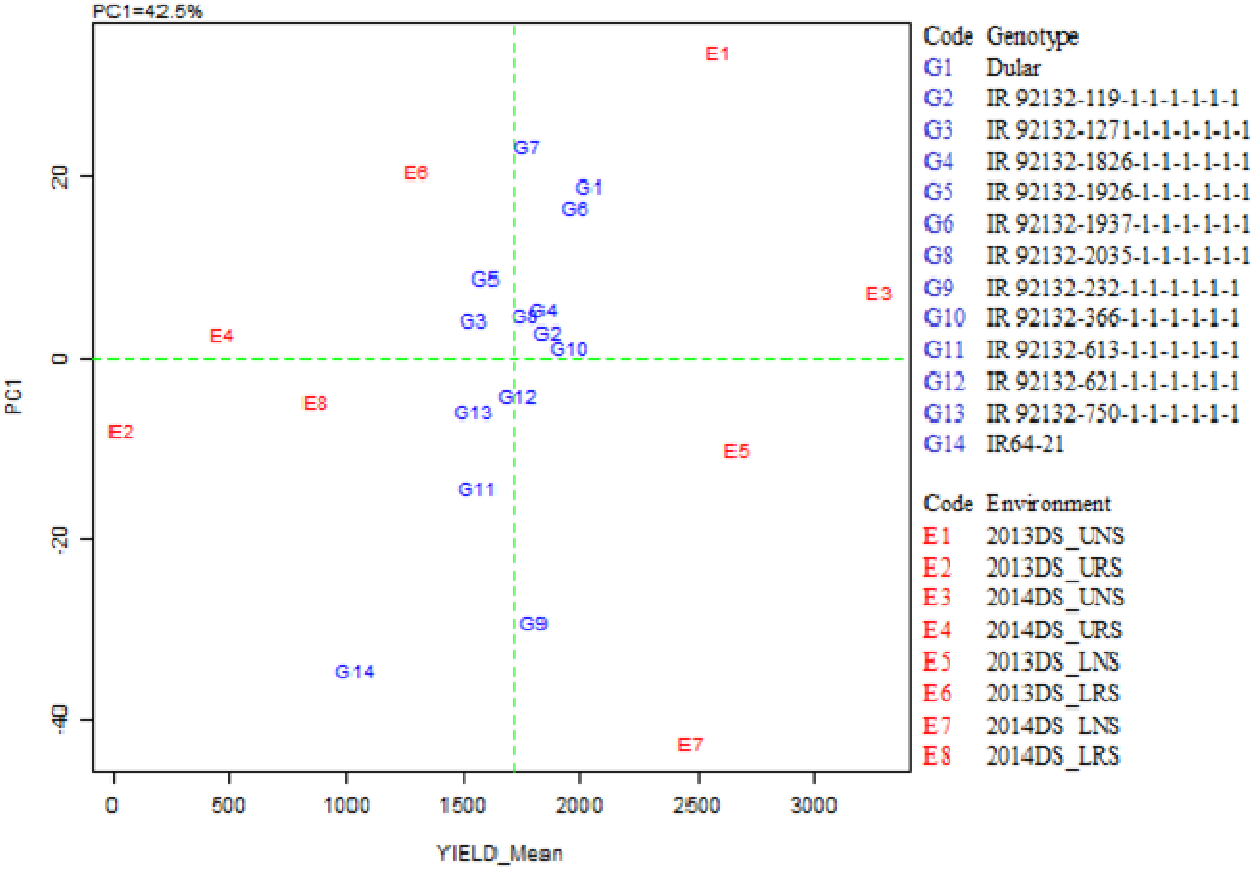

SUPPLEMEMTARY FIGURE 1| Yield stability plot from AMMI analysis. The vertical green line represents the mean yield across experiments and the horizontal green line indicates the IPCA value for the most stable yield across all environments.
