# Supplementary material for: Genetic Loci Governing Grain Yield and Root Development under Variable Rice Cultivation Conditions
Source: Front Plant Sci. 2017 Oct 16;8:1763. doi: 10.3389/fpls.2017.01763 (PMC5650699; doi:10.3389/fpls.2017.01763)
Supplement: Supplementary file 4 [file Image2.PDF]

Supplementary Material

Exploiting genetic loci enhancing grain yield and root development under variable cultivation conditions

Margaret Catolos[1,2], Nitika Sandhu[1], Shalabh Dixit[1], Noraziyah Abd Aziz Shamsudin[1,3], Elizabeth Naredo[1], Kenneth McNally[1], Amelia Henry[1], Ma. Genaleen Diaz[2] and Arvind Kumar[1]*

*** Correspondence:** Arvind Kumar: a.kumar@irri.org

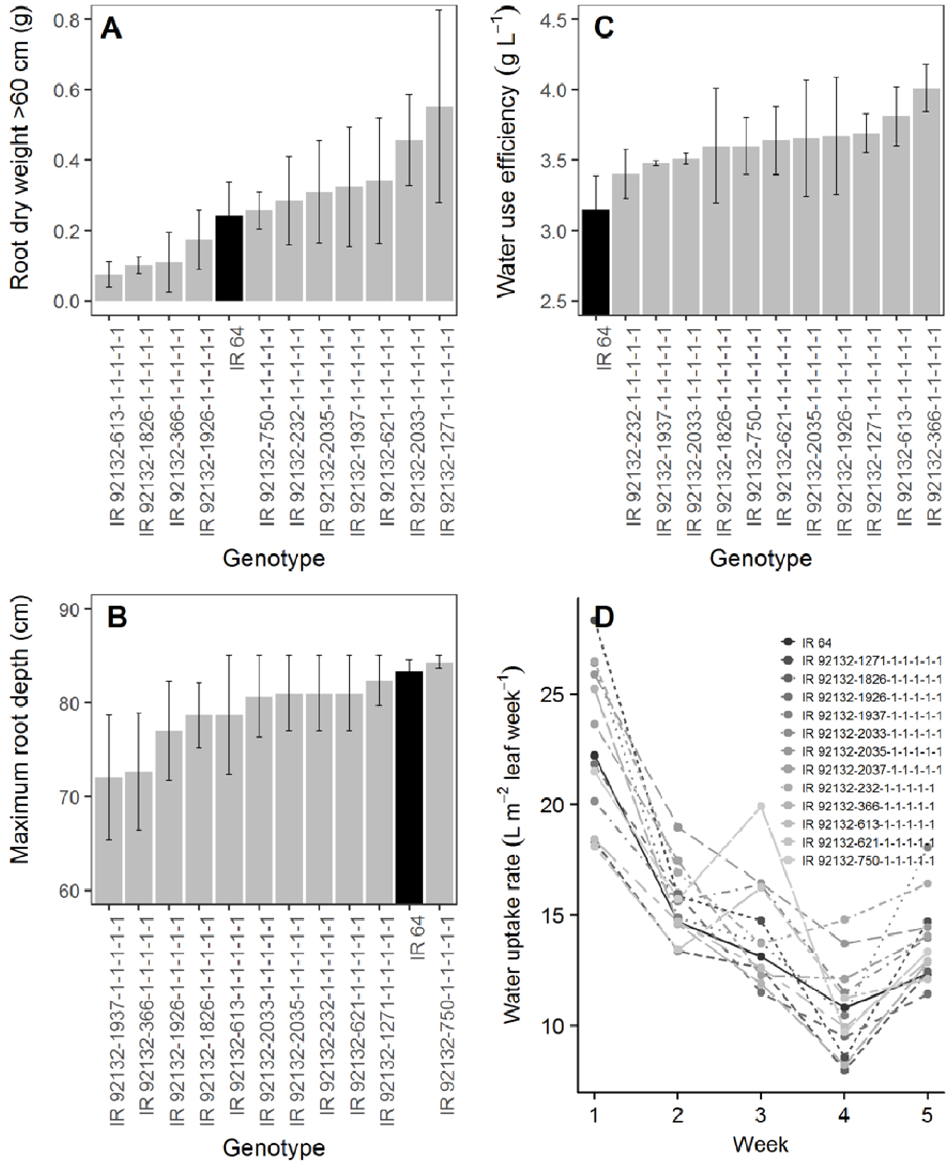

SUPPLEMEMTARY FIGURE 2| Greenhouse lysimeter experiment: Root growth and water uptake of the most stable, high-yielding genotypes of the IR64 x Dular population.
