# Supplementary material for: Genetic Loci Governing Grain Yield and Root Development under Variable Rice Cultivation Conditions
Source: Front Plant Sci. 2017 Oct 16;8:1763. doi: 10.3389/fpls.2017.01763 (PMC5650699; doi:10.3389/fpls.2017.01763)
Supplement: Supplementary file 5 [file Image3.PDF]

Supplementary Material

Exploiting genetic loci enhancing grain yield and root development under variable cultivation conditions

Margaret Catolos[1,2], Nitika Sandhu[1], Shalabh Dixit[1], Noraziyah Abd Aziz Shamsudin[1,3], Elizabeth Naredo[1], Kenneth McNally[1], Amelia Henry[1], Ma. Genaleen Diaz[2] and Arvind Kumar[1]*

*** Correspondence:** Arvind Kumar: a.kumar@irri.org

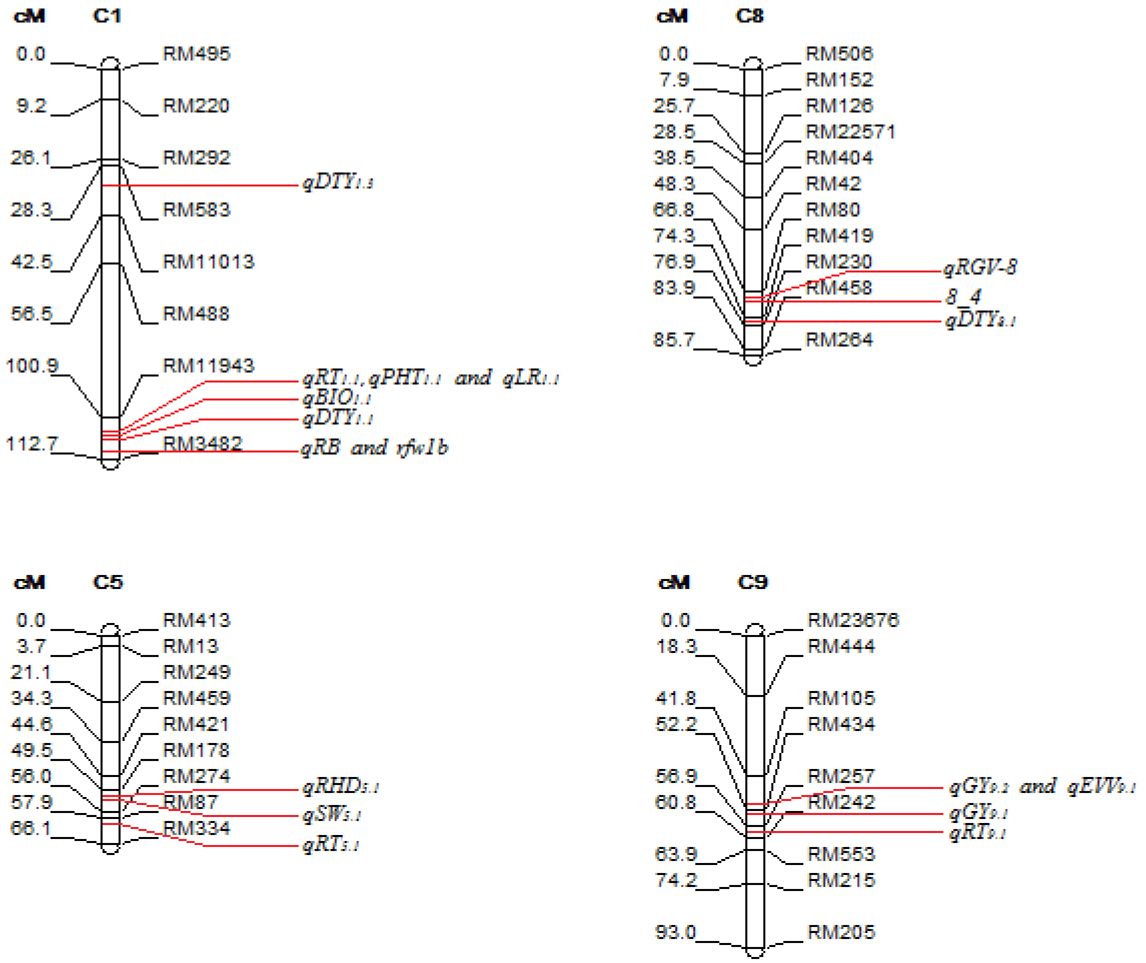

SUPPLEMEMTARY FIGURE 3| Linkage map of chromosomes 1, 5, 8, and 9 showing the position of known QTLs co-locating to the QTLs identified in the present study.
